# Regulation of the mammalian maternal-to-embryonic transition by eukaryotic translation initiation factor 4E

**DOI:** 10.1242/dev.190793

**Published:** 2021-06-25

**Authors:** Yan Li, Jianan Tang, Xu Ji, Min-Min Hua, Miao Liu, Lu Chang, Yihua Gu, Changgen Shi, Wuhua Ni, Jing Liu, Hui-juan Shi, Xuefeng Huang, Christopher O'Neill, Xingliang Jin

**Affiliations:** 1 Reproductive Medicine Center, The First Affiliated Hospital of Wenzhou Medical University, Wenzhou, Zhejiang Province, 325000, China; 2 NHC Key Lab of Reproduction Regulation, Shanghai Institute of Planned Parenthood Research, Fudan University, Shanghai, 200032, China; 3 Department of Pharmacology, School of Pharmaceutical Sciences, Nanjing Tech University, Nanjing, Jiangsu Province, 211816, China; 4 Reproductive Medical Center, Zhongshan Hospital, Fudan University, Shanghai, 200032, China; 5 Human Reproduction Unit, Sydney Center for Regenerative and Developmental Medicine, Kolling Institute for Medical Research, Sydney Medical School, University of Sydney, St. Leonards, New South Wales, 2065, Australia

**Keywords:** Cap-dependent translation, Early embryo development, Mammalian maternal embryonic transition, Transposon, Mouse

## Abstract

Eukaryotic translation initiation factor 4E (eIF4E) mediates cap-dependent translation. Genetic and inhibitor studies show that eIF4E expression is required for the successful transition from maternal to embryonic control of mouse embryo development. eIF4E was present in the oocyte and in the cytoplasm soon after fertilization and during each stage of early development. Functional knockout (*Eif4e*^−/−^) by *PiggyBac [Act-RFP]* transposition resulted in peri-implantation embryonic lethality because of the failure of normal epiblast formation. Maternal stores of eIF4E supported development up to the two- to four-cell stage, after which new expression occurred from both maternal and paternal inherited alleles. Inhibition of the maternally acquired stores of eIF4E (using the inhibitor 4EGI-1) resulted in a block at the two-cell stage. eIF4E activity was required for new protein synthesis in the two-cell embryo and *Eif4e*^−/−^ embryos had lower translational activity compared with wild-type embryos. eIF4E-binding protein 1 (4E-BP1) is a hypophosphorylation-dependent negative regulator of eIF4E. mTOR activity was required for 4E-BP1 phosphorylation and inhibiting mTOR retarded embryo development. Thus, this study shows that eIF4E activity is regulated at key embryonic transitions in the mammalian embryo and is essential for the successful transition from maternal to embryonic control of development.

## Abstract (precis)

**Summary:** Combined use of a *PB [Act-RFP]* transgenesis model, selective pharmacological inhibition and expression analyses verified the essential role of eIF4E in the transition from maternal to embryonic control of mouse development.

## INTRODUCTION

Reproduction in all metazoans requires the conversion of the terminally differentiated gametes into the totipotent cells of the early embryo. The earliest stages of embryo development are under the control of transcripts and proteins inherited from the gametes, primarily the oocyte. The transition from maternal to embryonic control of embryo development is accompanied by the generation of a new transcriptome and proteome. In mammals, the oocyte and zygote are transcriptionally inert until embryo genome activation (EGA) (Tadros and Lipshitz, 2009; Svoboda, 2018). In the mouse, new transcription is initiated in zygotes and definitive embryonic transcription begins in the two-cell embryo (Bouniol et al., 1995). This is accompanied by the degradation of almost all the maternally inherited transcripts (Abe et al., 2015). Understanding of the details of proteome reprogramming in the early mammalian embryo is also now emerging (Wang et al., 2010; Gao et al., 2017; Israel et al., 2019). Many proteins present within the oocyte are also detected in the zygote; however, a proportion are rapidly lost after fertilization (Gao et al., 2017). This is accompanied by the increased protein expression of many components of ubiquitin/proteosome protein degradation. A range of other proteins is present at higher levels in the zygote than in the oocyte and includes proteins associated with the citrate cycle pathway, glucan metabolism, lipid-binding proteins and fatty acid metabolism (Wang et al., 2010; Gao et al., 2017; Israel et al., 2019). This early round of new translation also includes components of the transcriptional machinery (Wang and Latham, 2000) and translational activity is required for the successful activation of the embryonic genome (Wang et al., 2001). After EGA, the new transcriptome is subject to a further round of translation; however, this does not result in a strong correlation between the RNA species generated at EGA and the cellular proteome until the morula and blastocyst stages (Gao et al., 2017; Israel et al., 2019).

The factors regulating translation in the mammalian embryo have not yet been analyzed in detail. In eukaryotes, more than 95% of proteins are synthesized through 5′-methylguanosine (m^7^G) cap-dependent mRNA translation (Gingras et al., 2001; Sonenberg and Hinnebusch, 2009). Eukaryotic translation initiation factor 4E (eIF4E) is generally considered the rate-limiting factor in translation from mRNA (Truitt et al., 2015). This protein recognizes and binds to the m^7^G cap moiety on mRNA within the cytoplasm (Culjkovic et al., 2005). It also participates in the formation of a multiprotein complex that also contains eIF4A and eIF4G (Sonenberg and Hinnebusch, 2009); the formation of this complex is a rate-limiting step in cap-dependent translation (Gingras et al., 1999).

eIF4E binding to mRNA cap structures is inhibited by a small family of eIF4E-binding proteins (4E-BPs) in their hypophosphorylated state (Haghighat et al., 1995). However, the phosphorylation of 4E-BP dissociates it from eIF4F, allowing the formation of the eIF4E complex and initiation of cap-dependent translation (Gingras et al., 2001; Sekiyama et al., 2015). Among these proteins, eIF4E-binding protein 1 (4E-BP1) is the most abundant and is phosphorylated at multiple sites by a phosphoinositide (PI) 3-kinase/protein kinase B (Akt)/mechanistic target of rapamycin kinase (mTOR)-dependent mechanism (Gingras et al., 2001). eIF4E function can also be regulated by phosphorylation at serine 209 (Ser209) by p38 mitogen-associated protein kinase 14 (Mapk14, also known as p38 MAPK) and the MAPK signal-integrated kinase (MNK) signaling pathway (Ueda et al., 2004; Li et al., 2010). The role of eIF4E phosphorylation is not clear because hypophosphorylated eIF4E can bind the mRNA caps and stimulate translation *in vitro* (Sonenberg and Hinnebusch, 2009; Hershey et al., 2012); nevertheless, phosphorylated eIF4E (p-eIF4E) is reported to enhance the translation of a subset of proteins (Furic et al., 2010).

In sea urchins, fertilization triggers dissociation of eIF4E from 4E-BP, allowing rapid recruitment of eIF4E into a high-molecular-mass complex. 4E-BPs are rapidly phosphorylated (Cormier et al., 2001) and degraded following fertilization, and this involves a rapamycin-sensitive mTOR signaling pathway (Salaün et al., 2003, 2005). In mammalian oocytes, temporal and spatial control of translation is regulated via an mTOR-eIF4F pathway (Susor et al., 2015). Although genetic studies have shown that *Eif4e*^+/−^ mice are viable (Truitt et al., 2015), the phenotype of *Eif4e-*null embryos has, to our knowledge, not yet been described in detail.

In this study, we used mouse *PiggyBac* (*PB*) transposon-induced transgenesis to produce a functional *Eif4e* knockout (KO). It showed that *Eif4e*^−/−^ was embryonic lethal, with lethality occurring around the time of implantation. eIF4E showed temporal and subcellular regulation throughout early embryo development and was required for successful embryonic development.

## RESULTS

### eIF4E and p-eIF4E in the mouse oocyte and preimplantation embryo

**Fig. 1.**
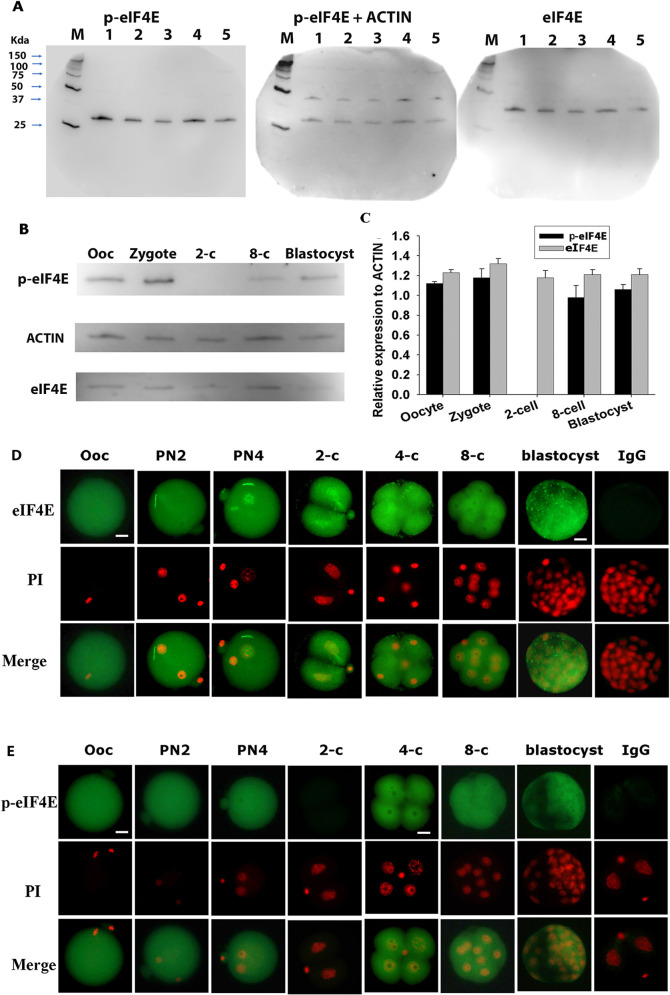
**eIF4E and p-eIF4E in mouse gametes and preimplantation embryos.** (A) Western blot analyses of p-eIF4E, actin and eIF4E. Lanes 1-5 indicate independent repeats of 50 zygotes for each lane. Images are representative gels of four replicates. (B) Representative images of three replicates of western blot analyses of p-eIF4E, eIF4E and actin in oocytes and preimplantation embryos. (C) Relative fold changes in p-eIF4E and eIF4E relative to actin in oocytes and preimplantation embryos. Each sample contained 50 oocytes or embryos. Mean±s.e.m. of the molecular weights of eIF4E, p-eIF4E and actin were 32.98±0.13, 28.05±0.16 and 40.82±0.5 kDa, respectively, determined by comparison with a prestained protein ladder. (D,E) Whole sections of oocytes and preimplantation embryos epifluorescent-stained for eIF4E (D) and p-eIF4E (E) and counter-stained with propidium iodine (PI). Images are representative of more than 30 embryos at each developmental stage. Negative controls were stained with non-reactive IgG and generated no signal at any developmental stage. Scale bars: 10 µm. M, molecular weight standards.

We first established a method for the simultaneous quantitative detection of eIF4E and p-eIF4E relative to the levels of actin in embryos using western blot. Clear signals for all three antigens could be detected in groups of 50 zygotes with little variability in signal strength between samples (Fig. 1A). A single band of eIF4E and p-eIF4E was observed in mouse oocytes during each stage of preimplantation development (Fig. 1B). The larger size of the eIF4E band compared with that of p-eIF4E may indicate a post-translational modification, such as sumoylation (Xu et al., 2010a,b). The detection level of both antigens was relatively stable relative to actin levels throughout each developmental stage, except for lower p-eIF4E during the two-cell stage (Fig. 1C). These antibodies were next used to assess the subcellular distribution of each antigen in embryos.

eIF4E antigen staining was evenly distributed across the oocyte; however, in the embryo it was characterized by accumulation within the nucleolus precursor bodies (NPB) present both in parental pronuclei in the zygotes and in two-cell stage embryos. This was not evident from the eight-cell stage embryo (Fig. 1D). p-eIF4E was evenly distributed across the cells in all stages, except the two-cell embryo, in which it was not detected (Fig. 1E). Unlike the native protein, p-eIF4E did not show an obvious accumulation within NPBs. This relatively pervasive presence of eIF4E and p-eIF4E in the oocyte and across early stages of embryo development is indicative of a likely role in these reproductive processes, whereas its differential localization patterns at key embryonic transitions may be indicative of active regulation.

### The developmental viability of *Eif4e*-deficient mice

**Fig. 2.**
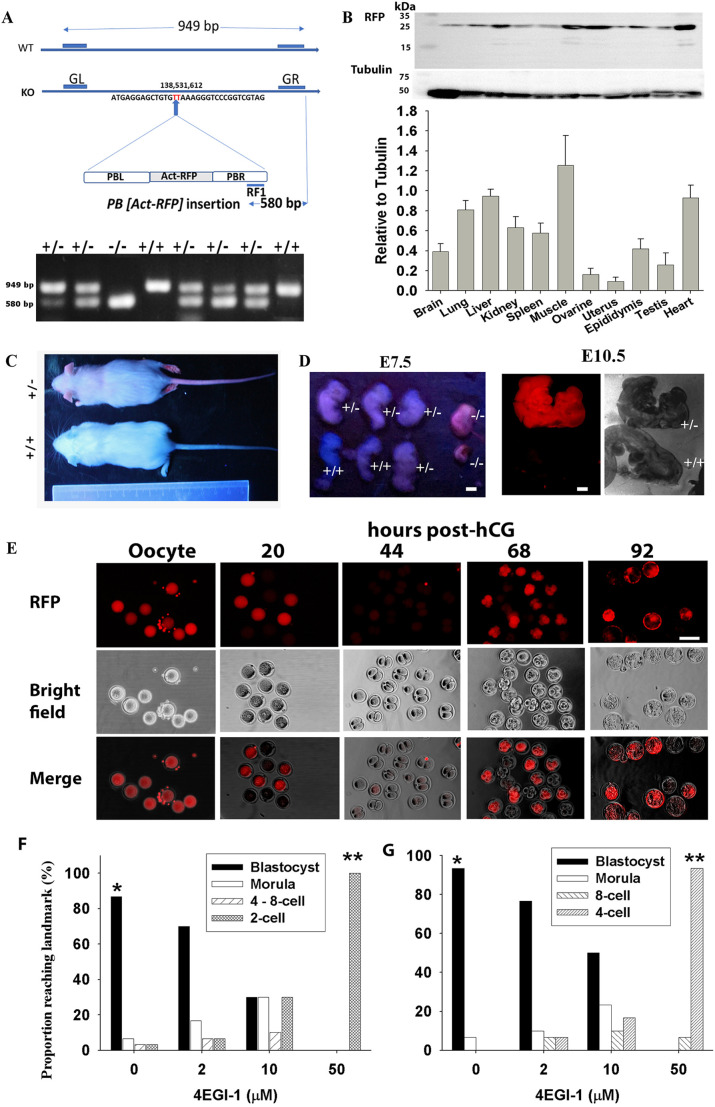
**Functional knockout of *Eif4e* with PB transposon and pharmacological inhibition during mouse early embryo development.** (A) Construct and gel electrophoresis analysis of the *PB [Act-RFP]* transposon and its insertion position in *Eif4e*. Mouse *Eif4e* (NC_000069.6) is localized at 138,526,191-138,559,696 on chromosome 3 (ENSMUSG00000028156). The *PB [Act-RFP]* transposon (insert number: 080429020-HRA) was positioned at the TTAA target site 138,531,612 of the second intron of *Eif4*e, as confirmed by sequencing analysis referring to mouse genome assembly GRCm38.p6 from the Genome Reference Consortium (https://www.ncbi.nlm.nih.gov/assembly/GCF_000001635.26/) (Fig. S1), which is relevant to 138,237,373 to GRCm39 (https://www.ncbi.nlm.nih.gov/assembly/GCF_000001635.27/). The GL and GR primers detected a 949 bp product representing wild-type *Eif4e*. The PB primer (RF1, CCTCGATATACAGACCGATAAAACACATGC) used to detect this insertion was localized on the right side of the *PB* transposon (PBR), which gave the 580 bp products. (B) Western blot analysis (top) and quantification of (bottom) of the expression and mean fold change of RFP relative to tubulin for 11 *Eif4e*^+/−^ mouse organs. Data are mean±s.e.m.; *n*=3. (C) RFP^−^ *Eif4e*^+/+^ and RFP^+^ *Eif4e*^+/−^ pups from *Eif4e*^+/−^ cross-mating. (D) RFP expression and genotyping in E7.5 and E10.5 embryos from *Eif4e*^+/−^ females. (E) RFP expression in oocyte and preimplantation developmental stages. At least 50 oocytes or embryos were analyzed for each development stage. (F,G) Effects of 4EGI-1 on preimplantation development *in vitro*. Data are representative of three independent replicates of either cultured hybrid zygotes (F) or two-cell embryos (G) in media dosed with 4EGI-1 for 96 h or 72 h, respectively. *P<0.001 (compared with the 4EGI-1 treatments), **P<0.001 (compared with the control and the lower doses 4EGI-1 treatments) (binary logistic regression analysis). Scale bars: 1 mm in D; 10 μm in E.

The mouse founders (*Eif4e*^+/−^) carried an insertion of the *PB [Act-RFP]* transposon (Table S1) into *Eif4e* (Fig. 2A). This transposon was identified to be uniquely located on chromosome 3, 138531612, by analysis of genomic sequencing against the mouse genome (mm38) (Fig. S1). The genotypes of offspring were analyzed by PCR (Fig. 2A). RFP protein was detected by western blot as a single band of the expected size of 27 kDa and displayed different expression levels across the 11 *Eif4e*^+/−^ mouse organs analyzed (Fig. 2B). Cross-breeding *Eif4e*^+/−^ mice produced *Eif4e*^+/+^ or *Eif4e*^+/−^ but no *Eif4e*^−/−^ progeny (Table 1). *Eif4e*^+/−^ pups were fluorescent red as a result of the expression of RFP present within the *PB [Act-RFP]* construct (Fig. 2C). The total number and the number per litter of *Eif4e*^+/−^ pups were smaller than the expected theoretical Mendelian ratios (MR) (*P*<0.01) (Table 1). At 4 weeks of age, the body weight of *Eif4e*^+/−^ pups was lower than that of their *Eif4e*^+/+^ littermates (*P*<0.01) (Table 1) for both sexes, but there was no significant difference between sexes within the same genotype. Several implantation sites had incomplete or inviable embryonic tissue at embryonic day (E) 7.5; for each site, the dissected embryonic tissue was from *Eif4e*^−/−^ embryos (Fig. 2D). By E10.5, only *Eif4e*^+/−^ and *Eif4e*^+/+^ embryos were present (Fig. 2D). These results show that *Eif4e*-null embryos are not capable of development beyond early gestation, whereas *Eif4e* hypomorphic mice were viable but had reduced postnatal growth rates.

**Table 1.**
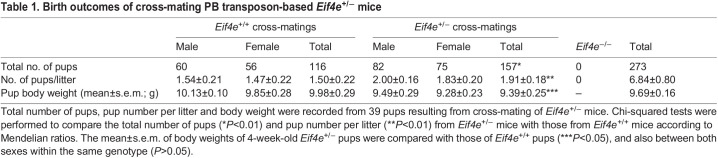
Birth outcomes of cross-mating PB transposon-based *Eif4e*^+/−^ mice

To gain insights into the likely causes of this loss of viability of *Eif4e*-null embryos, we next examined the expression of the RFP gene and early development rates of embryos derived from *Eif4e*^+/−^×*Eif4e*^+/−^ cross-mating. All oocytes collected from the reproductive tracts of *Eif4e*^+/−^ females were RFP positive (RFP^+^), indicating its premeiotic II expression (Fig. 2E). After fertilization, approximately half of the zygotes were RFP^+^, whereas no morphological two-cell stage embryos collected from the reproductive tract were RFP^+^ (Fig. 2E; Table 2; Table S2). In the four- to eight-cell and blastocyst stages, ∼69% of embryos were RFP^+^ (Fig. 2E; Table S2). These results suggest that the maternally inherited RFP is rapidly degraded upon fertilization and is then re-expressed soon after the activation of transcription from the new embryonic genome (i.e. embryonic genome activation). It is not clear why 50% of zygotes were still RFP^+^. This may simply reflect variability in the rates of degradation between cells; however, if it is dependent upon the status of the maternally inherited gene, then it may indicate either that the ACT-RFP allele is transcribed during the earliest rounds of low-level transcription that occur in the zygote or that differing rates of RFP degradation occur depending on the genetic background of the oocyte. Thus, this result requires further investigation.

**Table 2.**
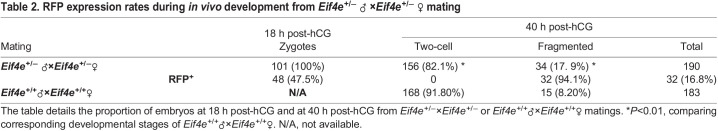
**RFP expression rates during *in vivo* development from *Eif4e*^+/−^** ♂ **×*Eif4e*^+/−^** ♀ **mating**

Approximately 18% of embryos resulting from *Eif4e*^+/−^×*Eif4e*^+/−^ cross-mating were retarded or significantly fragmented when collected 40 h post human chorionic gonadotropin (hCG) administration and, of these, 94% were RFP^+^ (Table 2) (it was not possible to genotype these cells reliably). This rate of fragmentation was significantly higher than observed in embryos collected from wild-type crosses (Table 2). Culturing the two-cell embryos for a further 24 h resulted in ∼50% developing to the four- to eight-cell stage and, of these, 66.7% were RFP^+^. Individual genotyping of these four- to eight-cell-stage embryos showed that 67.7% were either heterozygous or homozygous; thus, by this stage, ∼98% of embryos carrying ACT-RFP had regained the capacity to express RFP. By contrast, only 53% of embryos that were retarded after 24 h culture (i.e. remaining at the two-cell stage) were RFP^+^, whereas genotyping showed that 83% of the retarded embryos were either heterozygous or homozygous (*P*>0.05) (Table 3). Genotyping of individual blastocysts cultured for 96 h from the zygote stage did not show any skew from the expected Mendelian distribution (*P*>0.05). All heterozygous or homozygous embryos were RFP^+^, whereas no wild-type embryos expressed RFP^+^ (Table 3). The results showed that eIF4E is a maternal product that is lost from the embryo progressively after fertilization. It then becomes progressively re-expressed from the late two-cell stage, at which definitive transcription from the embryonic genome is known to be initiated (Flach et al., 1982). This analysis also showed that the rapid degradation of maternal stores and the reactivation of RFP expression allow RFP detection to serve as a reliable marker of embryos past the eight-cell stage that carry the mutant allele.

**Table 3.**
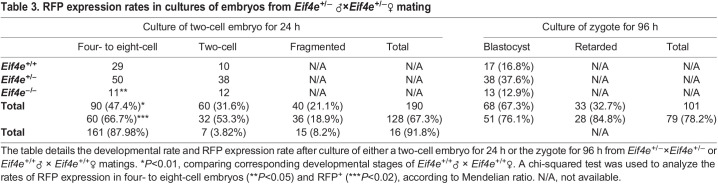
**RFP expression rates in cultures of embryos from *Eif4e*^+/−^** ♂**×*Eif4e*^+/−^**♀ **mating**

Analysis of the distribution of embryos between each of the expected genotypes showed that, at the four- to eight-cell stage, fewer *Eif4e*^−/−^ embryos were detected than expected (*P*<0.02) (Table 3), whereas, at the blastocyst stage, this skew did not achieve significance for the sample size available (*P*>0.05). A number of embryos were retarded in development (i.e. having not reached the expected developmental landmark of either four to eight-cells 48 h post hCG or normal morphological blastocysts after 96 h culture) and, of these, 94% (*P*<0.05 compared with the expected 75%) and 85% (*P*>0.05) were RFP^+^, respectively (Table 3). The results showed that, although there may be some loss of *Eif4e*^−/−^ embryos prior to the blastocyst stage, most embryos lacking the functional gene are able to form morphological blastocysts.

Analysis of a public database (Deng et al., 2014) that allowed us to determine whether *Eif4e* expression was from the maternal and paternal inherited alleles showed that, in the zygote and early two-cell embryo, all transcripts of maternal origin were consistent with these transcripts originating in the oocyte (Fig. S2A). By the late two-cell stage, when definitive new transcription from the embryonic genome has occurred, transcripts of paternal origin were seen for the first time, and this source had increased proportionally by the four-cell stage. Thereafter, there was approximately equivalent contributions from both alleles. Analysis of individual cells showed that, during the late two-cell stage, biallelic expression was always evident whereas, as development progressed, the incidence of apparently random monoallelic expression increased to ∼60% of cells and involved both alleles (Fig. S2B). This analysis supports a conclusion that the early embryo inherits a maternal store of *Eif4e* transcripts and that new transcription from both alleles occurs following zygotic genome activation in the late two-cell embryo.

The significant stores of eIF4E observed in the gametes and carried over into the zygote and the high rate of embryonic loss by the two-cell stage point to a possible role for the protein prior to the onset of definitive embryonic genome activation. The presence of the gametic stores did not allow this question to be explored with this genetic model; therefore, the effect of selective pharmacological inhibition of eIF4E (using 4EGI-1; K_D_=25 µM; Sekiyama et al., 2015) on this early stage of embryo development was assessed. 4EGI-1 blocks eIF4E binding to eIF4G, preventing the formation of active complexes (Sekiyama et al., 2015). Embryos collected at either the zygote or two-cell stage were cultured in media supplemented with a dose range of 4EGI-1. All doses caused a significant developmental block (*P*<0.001). At the highest dose, 4EGI-1 caused a complete developmental block of zygotes (blocked at the two-cell stage) and of two-cell embryos (blocked at the four-cell stage) (Fig. 2F,G). By contrast, the presence of 50 µM 4EGI-1 did not affect fertilization by either *in vitro* fertilization (IVF) or intracytoplasmic sperm injection (ICSI) (Table 4); however, continued culture in the presence of 4EGI-1 did prevent development of the resulting zygotes to the morphological two-cell embryo. When fertilized eggs derived in 4EGI-1 medium were transferred into control medium, the blastocyst formation rate was rescued compared with controls (Table 4). This indicates that eIF4E activity from gametic stores is not required for fertilization, but is necessary for normal processes involved in the transition from maternal to embryonic control of development.

**Table 4.**
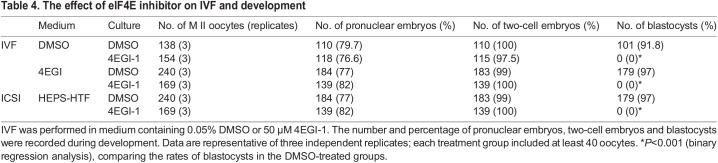
The effect of eIF4E inhibitor on IVF and development

**Fig. 3.**
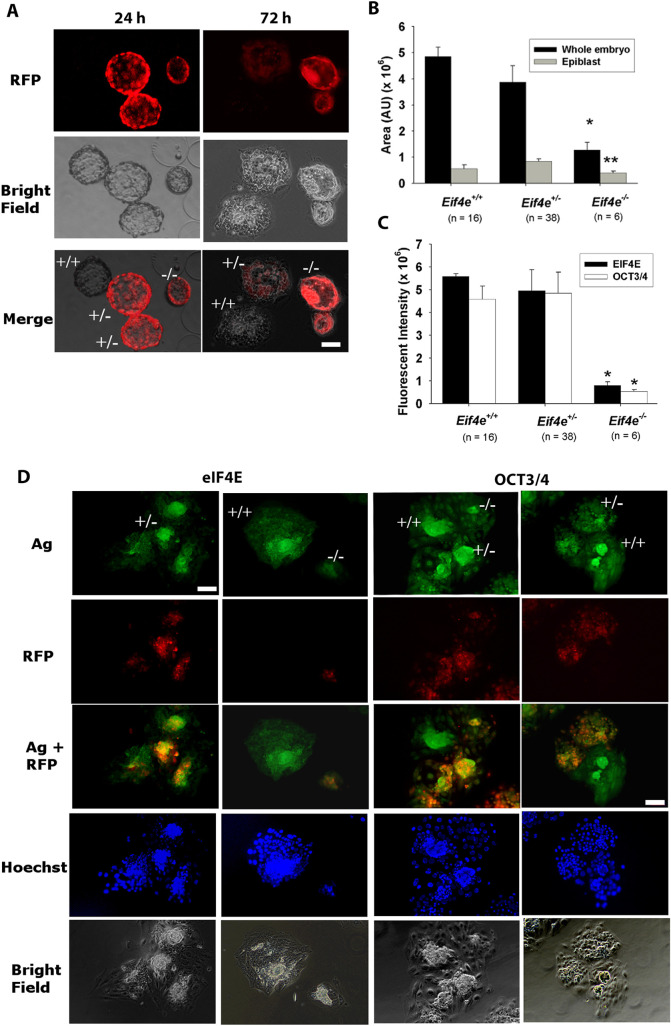
**Outgrowth viability of blastocysts from *Eif4e*^+/−^ cross-mating.** (A-D) Morphological blastocysts derived from the culture of two-cell embryos from *Eif4e*^+/−^ cross-matings were cultured for 96 h. (A) RFP expression in outgrowth embryos labeled with their *Eif4e* genotype. (B) Area (AU) of whole embryos and epiblasts resulting from *Eif4e*^+/−^ cross-matings. (C) Fluorescence intensity (AU) of eIF4E and OCT3/4 staining of whole embryos after 96 h of culture. Data are representative of 58 embryos from five matings (Table 5). *P<0.001, **P<0.01 (univariate analysis of variance, compared with the corresponding measurements for other genotypes). (D) Whole-session epifluorescent staining of outgrowths for OCT3/4, eIF4E, RFP and Hoechst 33342. Scale bars: 20 µm. Ag, antigen (i.e. eIF4E or OCT3/4).

Given that *Eif4e*^−/−^ embryos had some capacity to develop to morphological blastocysts but that no viable *Eif4e*-null embryos were detected by E7.5, we assessed the capacity of *Eif4e*^−/−^ blastocysts to form normal outgrowths with a pluripotent epiblast *in vitro*. This showed that *Eif4e*^−/−^ blastocysts commonly had reduced expansion and either failed to produce outgrowths or produced very poor outgrowths that were smaller in size (Fig. 3A,B) and generally failed to produce an Oct3/4^+^ pluripotent epiblast compared with heterozygous or wild-type embryos (Fig. 3C,D; Table 5). The results suggest that the actions of new eIF4E stores produced during embryo genome activation are required for the formation of the earliest cell lineages within the embryo. The failure to form a normal pluripotent epiblast in the implanting embryo is likely to be a consequence of deficiencies in a range of proteins resulting from reduced protein translational activity in *Eif4e*-deficient embryos.

**Table 5.**
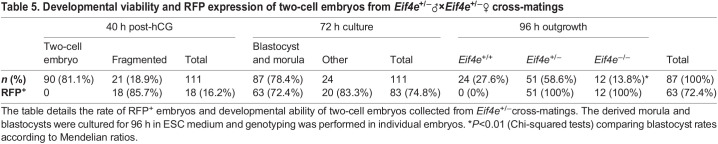
**Developmental viability and RFP expression of two-cell embryos from *Eif4e*^+/−^**♂**×*Eif4e*^+/−^**♀ **cross-matings**

### The effect of EIF4E on embryonic protein synthesis

**Fig. 4.**
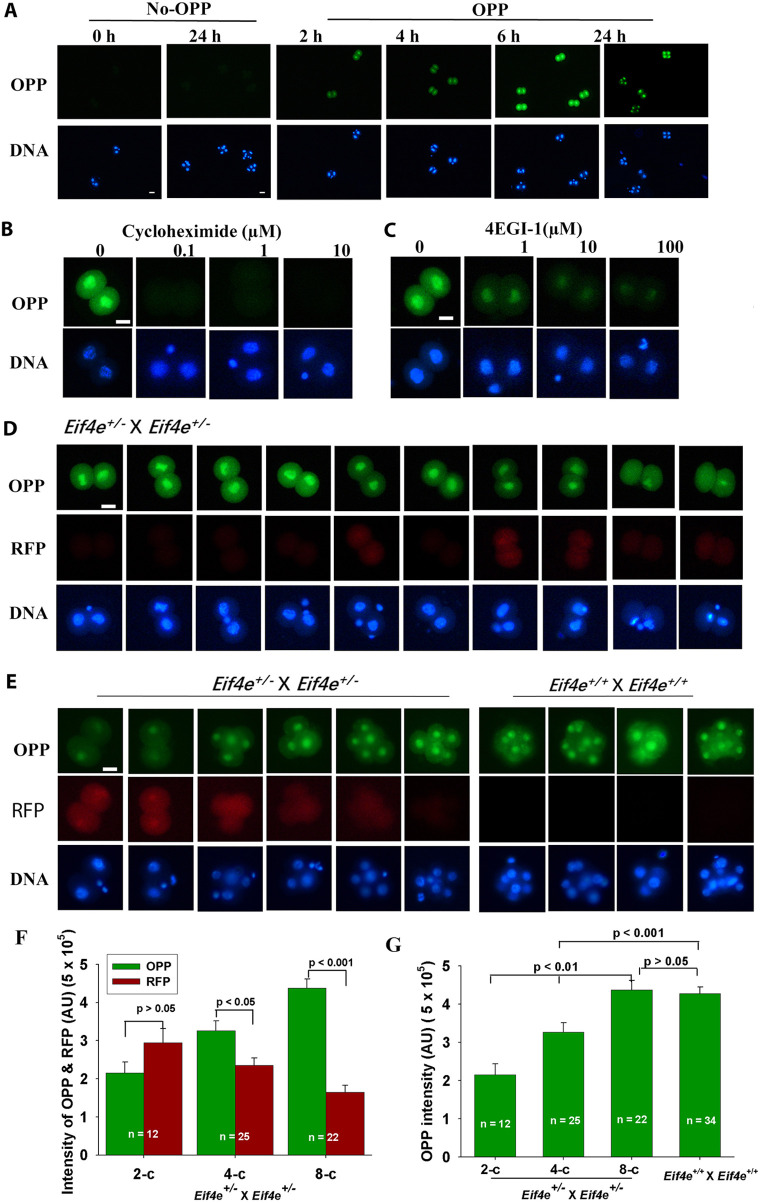
**Effects of eIF4E on embryonic translation.** (A) Whole-session images representative of fluorescence changes in wild-type two-cell embryos treated either without or with 37.5 µM OPP. There were at least ten embryos in each treatment group. (B,C) Two-cell embryos were treated with either cycloheximide (B) or the inhibitor 4EGI-1 (C) for 6 h. Images are representative of three independent replicates for ten embryos for each treatment. (D) Two-cell embryos were collected from *Eif4e*^+/−^×*Eif4e*^+/−^ cross-mated females and treated with 37.5 µM OPP for 6 h. The images are representative of a total of 60 embryos from three independent replicates. (E-G) One-cell embryos were collected from *Eif4e^+/−^*×*Eif4e^+/−^* cross-mated females, cultured for 42 h, and then treated with 37.5 µM OPP for 6 h. Images are representative of results from three independent replicates. Embryos from *Eif4e*^+/+^×*Eif4e*^+/+^ cross-mated females were used as controls. (E) Representative images of OPP, RFP and DNA staining of a total of 60 embryos collected from the two mating strategies described in D. (F) OPP and RFP staining intensity at each developmental stage of embryos from *Eif4e*^+/−^×*Eif4e*^+/−^ cross-matings after 42 h culture and then 6 h of OPP treatment. (G) OPP staining intensity in embryos from *Eif4e*^+/−^×*Eif4e*^+/−^ cross-matings compared with embryos from *Eif4e*^+/+^×*Eif4e*^+/+^ cross-matings (all of which developed to the eight-cell stage). Data are mean+s.e.m. (univariate analysis of variance). Scale bars: 10 μm.

To test whether *Eif4e* influenced the general translational activity within the embryo, we used the Click-iT^®^ Plus OPP Protein Synthesis Assay Kit. This demonstrated that new protein synthesis in mouse two-cell-stage embryos displayed a time-dependent increase in the incorporation of *O*-propargyl-puromycin (OPP) with the duration of incubation in the substrate reagent *in vitro* (Fig. 4A). This incorporation was blocked by the protein synthesis inhibitor cycloheximide (Schneider-Poetsch et al., 2010) (Fig. 4B) and the Eif4e inhibitor 4EGI-1 (Fig. 4C) in a dose-dependent manner.

Protein expression was also detected in two-cell embryos from *Eif4e*^+/−^ cross-mating that were treated with OPP for 6 h (Fig. 4D). The expression level differed between individual two-cell embryos, with the highest level of expression occurring in embryos that showed little RFP (Fig. 4D). Furthermore, for one-cell-stage-derived embryos cultured *in vitro* for 42 h, the level of translation was negatively associated with the level of RFP^+^ expression and the capacity of embryos to meet their expected developmental landmark (i.e. eight-cell stage) (Fig. 4E). Embryos that showed developmental delay with arrest prior to the expected eight-cell stage had the highest levels of RFP and a corresponding lower capacity for translation, as assessed by OPP-staining intensity (Fig. 4E,F). The overall level of protein expression was lower in embryos from *Eif4e*^+/−^ cross-mating than in embryos derived from *Eif4e*^+/+^ mating, but this was solely due to embryos that had retarded development and were likely to be either homozygous or heterozygous for the transgene, based on their high levels of RFP expression. Embryos from *Eif4e*^+/−^ crosses that achieved their expected developmental landmark of the eight-cell stage had significantly lower levels of RFP compared with retarded embryos and had similar levels of translation (OPP expression) as equivalent embryos from *Eif4e*^+/+^ crosses (Fig. 4G). However, the Click-iT method requires that embryos be cultured for varying periods *in vitro* and does not allow the assessment of any potential interactions between the genetic model and any adverse effects of the culture procedures. It was not possible to genotype embryos after the protein synthesis assays and, thus, this approach did not allow us to assess definitely changes in levels of protein synthesis relative to transgene dosage in individual embryos. The results show that embryos undergo active protein synthesis that is dependent upon eIF4E activity and that embryos derived from *Eif4e*^+/−^ crosses have variable, but generally lower, levels of protein synthesis compared with embryos derived from wild-type crosses.

### Regulation of EIF4E in the early embryo

**Fig. 5.**
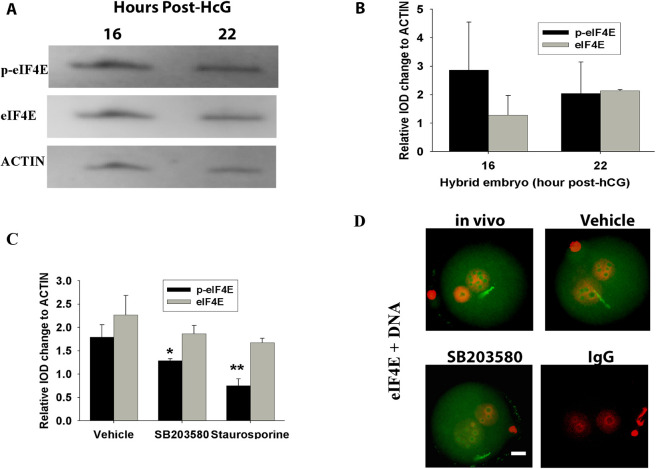
**Regulation of eIF4E and p-eIF4E.** (A) Western blots of eIF4E, p-eIF4E and actin expression in hybrid embryos (C57BL/6×CBA/He) at 16 and 22 h post-HCG. (B,C) Analysis of eIF4E and p-eIF4E expression changes relative to actin in hybrid embryos (C57BL/6×CBA/He) at 16 and 22 h post-HCG (B) and in hybrid embryos (C57BL/6×CBA/He) 16 h post-hCG treated for 4 h with either 10 µM SB203580 or 25 µM staurosporine (C). Data are representative of three independent replicates and each treatment had 50 embryos; *P<0.05, **P<0.01, compared with vehicle (univariate analysis of variance). (D) Whole-session immunolocalized images of eIF4E in hybrid embryos (C57BL/6×CBA/He) at 16 h post-hCG treated for 4 h with 10 µM SB203580 compared with vehicle. Data are representative of three independent replicates and each treatment group contained ten embryos. Scale bar: 10 µm.

A characteristic feature of the localization of the eIF4E antigen was its significant accumulation within the NBPs (Fig. 1C). Western blots showed similar levels of eIF4E and pSer209-eIF4E in the pronuclear (PN) 3 (16 h post hCG) and PN5 zygote (22 h post hCG) (Fig. 5A,B). Treatment of zygotes from 16 h post hCG with either 25 nM staurosporine (a broad-spectrum protein kinase inhibitor with some preference for protein kinase C) or 10 µM SB203580 (a selective p38 MAPK inhibitor) (Jin and O'Neill, 2014) reduced the levels of eIF4E phosphorylation, but did not affect the total eIF4E levels (Fig. 5C) in the resulting PN5 zygotes. Immunolocalization showed that SB203580 did not affect the subcellular localization of eIF4E staining, with accumulation within the NPBs still evident after this treatment (Fig. 5D). These results showed not only that the maintenance of maternal stores and NBP localization of eIF4E over this period of development were independent of the actions of a range of protein kinases, but also that maintenance of cellular levels of pSer209-eIF4E was dependent of the actions of kinases, including p38 MAPK.

**Fig. 6.**
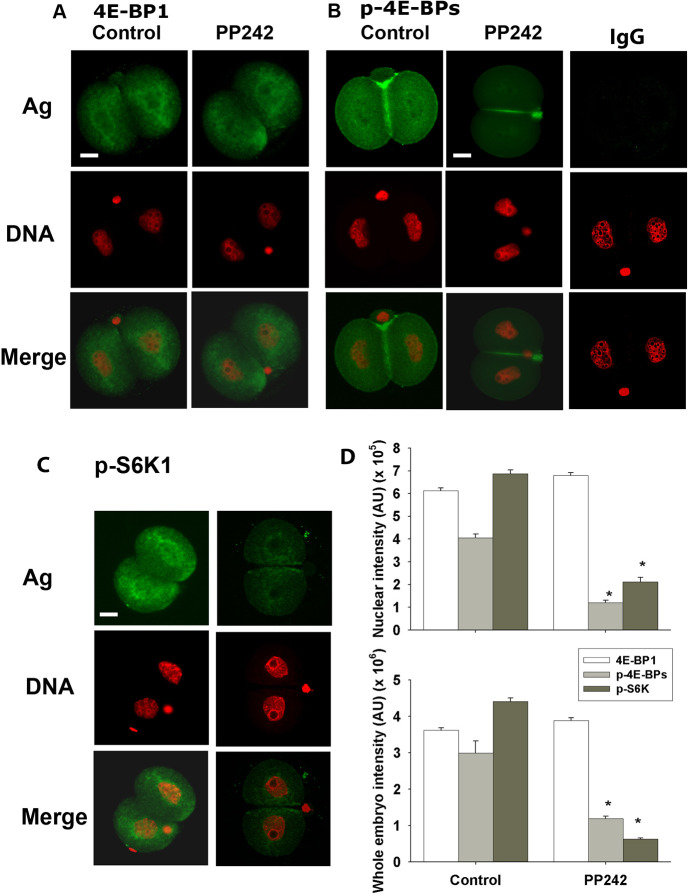
**mTOR-related 4E-BP1 and S6K1 signaling in mouse two-cell embryos.** (A-C) Representative confocal images of 4E-BP1 (A), p-4E-BPs (B) and p-S6K1 (C) in two-cell embryos derived from zygotes after treatment with 100 nM PP242. (D) Fluorescence intensity for each antigen in the nuclei and whole embryos. Data are representative of three independent replicates and each treatment group included at least ten embryos. Data are mean±s.e.m. *P<0.001 (univariate analysis of variance). Scale bars: 10 µm.

**Fig. 7.**
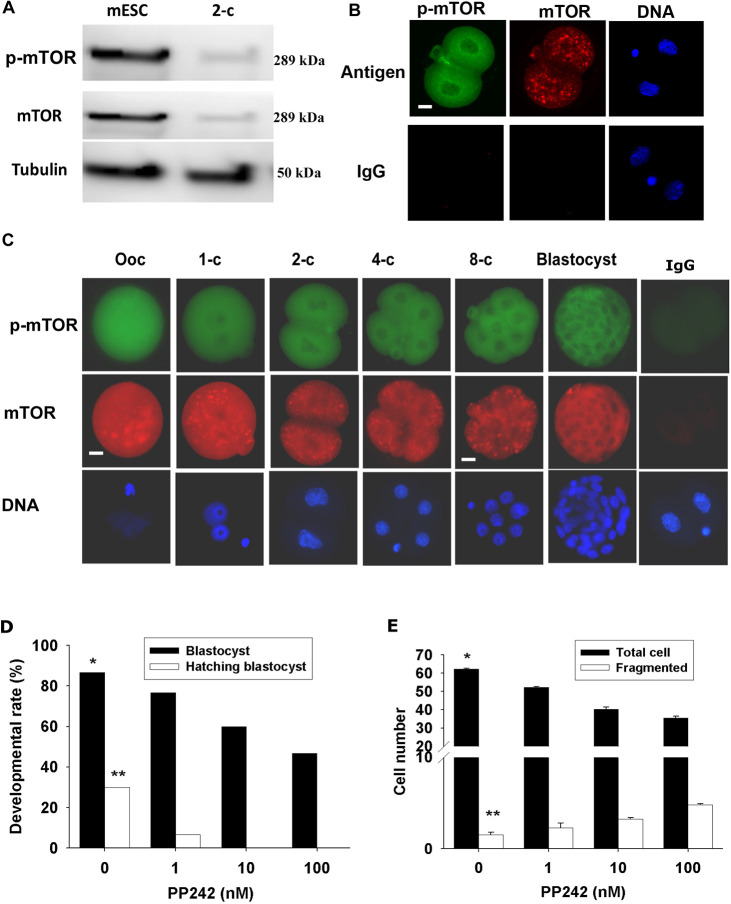
**mTOR and p-mTOR in mouse preimplantation embryos.** (A) Western blots of p-mTOR and mTOR in D3 mouse embryo stem cells (harvested in mLIF- and 10% FBS-added DMEM medium) and mouse two-cell embryos collected from oviducts. Protein from 1×10^6^ cells and 50 embryos were loaded. (B,C) Confocal immunostaining of p-mTOR, mTOR and Hoechst 33342 in a two-cell embryo (B) and epifluorescent immunostaining of p-mTOR, mTOR and Hoechst 33342 DNA in preimplantation embryos (C). Representative images of three independent replicates with ten embryos analyzed for each developmental stage. (D,E) Effects of PP242 on the rate of blastocyst formation and blastocyst hatching (D), and the number of total and fragmented cells in the formed blastocysts (E). Data are representative of three independent replicates. Each treatment included at least 20 embryos in each replicate. *P<0.001, **P<0.01 (univariate analysis of variance). Scale bars: 10 µm.

Another striking feature of the dynamics of the protein was the failure to detect the Ser209 p-eIF4E antigen within the two-cell embryo. This was surprising given the evidence that inhibition of eIF4E in the two-cell embryo caused a complete developmental block. Although phosphorylation has some control functions in somatic cells, it not clear whether it is a primary regulator of protein function. By contrast, 4E-BP1 is known to act as a translation repressor protein and, in its hypophosphorylated state, acts as a negative regulator of eIF4E-RNA complex formation (Gingras et al., 2001). 4E-BP1 is a substrate for mTOR and its action reverses this repressor activity, allowing eIF4E to undergo normal binding with m^7^G cap mRNA (Sekiyama et al., 2015). Immunolocalization showed 4E-BP1 to be present throughout the cytoplasm and nucleoplasm of the two-cell embryo and to be enriched at the nuclear periphery and perinuclear regions, whereas its level was restricted within NBPs (Fig. 6A). The phosphorylated form (pT45 4E-BP1+2+3) was also widely distributed across both the cells of the two-cell embryo. Its staining was characterized by a striking enrichment at the cleavage furrow (Fig. 6B). mTOR and Ser2448 p-mTOR were present throughout the two-cell embryo and in every other stage of preimplantation embryo development (Fig. 7A-C). Treatment of embryos with a selective inhibitor of mTOR (PP242; IC_50_=8 nM) (Apsel et al., 2008) had no effect on the level or localization of 4E-BP1 in the two-cell embryo, but caused a substantial loss of the pT45 4E-BP signal across the cells (Fig. 6A,B,D). Treatment of embryos with this drug also caused a dose-dependent retardation of embryo development (Fig. 7D,E). S6K1 is another important phosphorylation target of mTOR and the inhibition of its phosphorylation (pT389-S6K1) by PP242 served as a control, demonstrating the selectivity of the actions of this drug (Fig. 6C,D). Thus, we concluded that the activity of mTOR results in the phosphorylation of 4E-BP1 in the two-cell embryo, a result expected to favor eIF4E activity (Fig. 6); we also concluded that mTOR activity is necessary for normal embryo development in the mouse.

## DISCUSSION

PB-mediated transgenesis provides a highly efficient strategy for generating mice with a loss of gene function while allowing simultaneous monitoring of the transgene by the presence of RFP fluorescence (Chang et al., 2019). This model was used to disrupt the normal *Eif4e* gene by the insertion of the transgene within intron 2 of *Eif4e*. *Eif4e*^−/−^ embryos, resulting from *Eif4e*^+/−^ cross-mating, were inviable, with lethality occurring soon after embryo implantation: they were capable of development to morphological blastocysts, but these failed to show blastocyst outgrowth reliably and did not form a pluripotent epiblast. *Eif4e*^+/−^ embryos were viable, but resulted in progeny with a lower postnatal body weight.

The survival of *Eif4e*^−/−^ embryos to the blastocyst stage was possibly due to the persistence of maternal protein within the early embryo. Analysis of RFP expression showed that protein was carried over from the oocyte. The rapid loss of RFP after fertilization likely reflects a combination of the transcriptionally inert state of the early zygote and the onset of proteolytic degradation of many maternally inherited proteins after fertilization. However, the kinetics of RFP degradation may not reflect that of native eIF4E because there are many aspects of individual protein structure that influence these kinetics (Grumati and Dikic, 2018; Tsukamoto and Tatsumi, 2018; Toralova et al., 2020). It is also possible that the kinetics of protein degradation vary depending on whether embryos are collected directly from the reproductive tract or are cultured *in vitro*. Given the nature of the experimental design, it was not possible to control for these variables in this study.

The loss of RFP from the zygote facilitated the identification of its new expression initiated after definitive EGA and this occurred from both maternally and paternally inherited alleles of eIF4E (Yanagiya et al., 2012). This is consistent with the outcome of the analysis of allelic expression patterns of gene transcripts, which revealed the presence of maternally inherited transcripts in the zygote with new transcription from both alleles occurring after zygotic genome activation at the late two-cell stage. The complete developmental block of either zygotes or two-cell embryos caused by treatment with a selective eIF4E inhibitor indicated that the stores carried over from the oocyte were necessary for development prior to EGA. Given the expression of eIF4E from both alleles at EGA, it was interesting that *Eif4e*^+/−^ embryos developed normally past the implantation stage. This result is consistent with observations that mammalian cells express levels of eIF4E that are in excess of those required for normal cell function. Furthermore, *Eif4e*^+/−^ somatic cells show no apparent change in global protein synthesis (Truitt et al., 2015; Piserà et al., 2018). It could also be a result, in part, of the relatively high incidence of apparently random monoallelic expression of *Eif4e* within individual cells. However, *Eif4e*^+/−^ progeny had a smaller postnatal body weight, which indicates that some haploinsufficiency occurred in the heterozygous state, suggesting that haploinsufficiency was influenced by some unexpected complexity in the interactions between the gamete carrying the null allele and the maternal genotype.

This genetic and pharmacological evidence for the necessary actions of eIF4E at two crucial transitions during early embryo development (i.e. at the time of onset of EGA and normal epiblast formation) is consistent with similar discoveries in non-mammalian model species, such as sea urchins (Salaün et al., 2003, 2005), zebrafish (Fahrenkrug et al., 1999) and *Drosophila* (Hernández et al., 2005). The pervasive detection of both eIF4E and its phosphorylated form across all preimplantation stages of embryo development also supports its important roles in development. The analysis of translation in the early embryo was consistent with the expected roles of eIF4E in translation initiation. However, the current study did not determine which products of translation are required at the stages crucial for further development. The developmental blocks resulting from the early pharmacological inhibition of eIF4E may reflect the need for the translation of crucial components either of the mitotic machinery, such as the cyclins (Groisman et al., 2002; Culjkovic et al., 2005), or of the transcriptional apparatus required for EGA. For example, new protein synthesis of activating transcription factor (ATF1) is required for zygote maturation (Jin and O'Neill, 2014). Thus, this question requires further detailed analysis.

eIF4E is not normally present with nucleoli of somatic cells (Osborne and Borden, 2015); its accumulation within NPBs of the zygote is therefore interesting. NPBs are not considered to be functional nucleoli in the zygote. They lack fibrillar centers and the granular and dense fibrillar components seen in functional nucleoli. Furthermore, they do not appear to undertake ribosome production (Fulka and Langerova, 2014; Aguirre-Lavin et al., 2012). Nevertheless, NPB function is essential for normal embryonic development, indicating non-canonical roles for these structures (Fulka and Langerova, 2014). Their primary role appears to be related to the reprogramming and reorganization of centromeres and pericentric satellites into chromocenters (Probst and Almouzni, 2011). Thus, analysis of the role of eIF4E in the reprogramming of chromatin structure and organization in the zygote will be of interest.

Incorporation of eIF4E into the complexes required for the initiation of translation is regulated by its phosphorylation as well as through binding of inhibitory proteins (Šušor et al., 2008). eIF4E phosphorylation occurs in somatic cells via an MNK-mediated pathway (Wang et al., 1998; Shveygert et al., 2010; Piserà et al., 2018), and we show that p38 activity was required to maintain p-eIF4E levels in zygotes. Interestingly, although p-eIF4E was present in most stages of development tested, it was not detected in two-cell embryos; it has also been shown that 4E-BP1 becomes dephosphorylated in the porcine embryo after fertilization (Šušor et al., 2008). The roles of eIF4E phosphorylation are not yet fully defined. It is not required for normal cell growth or development in some models (Ueda et al., 2004), although it does appear to enhance translation from some mRNA species (Furic et al., 2010), indicating that the dynamic changes in eIF4E phosphorylation around the time of EGA warrant further investigation.

The binding of 4E-BP1 to eIF4E can block phosphorylation of eIF4E (Wang et al., 1998; Parra-Palau et al., 2003). The interaction of 4E-BP1 with eIF4E is, in turn, negatively regulated by 4E-BP1 phosphorylation via a PI3-kinase/AKT/mTOR signaling pathway (Ryu and Kim, 2017). Autocrine trophic ligands activate the PI3K/AKT pathway soon after fertilization and this activity is essential for the normal development and survival of the early embryo (Li et al., 2007; O'Neill, 2008; Jin et al., 2009). Here, we show that mTOR and its phosphorylated form are also present in the two-cell embryo. Inhibition of mTOR activity inhibited phosphorylation of 4E-BP1 in the zygote and also blocked embryo development. The results indicate that one role for autocrine trophic signaling pathways in the early embryo is to foster the activity of eIF4E.

This study shows that eIF4E, an essential component of the translation initiation complex, is a crucial maternal-effect product that is required for the development of the early mammalian embryo. Its presence at the crucial embryonic transitions of EGA and the first rounds of cellular differentiation places it as a key regulator of the transition of maternal-to-embryonic control of development. Thus, detailed analysis of the factors regulating translation in the embryo is an essential precondition to understanding the normal development of the mammalian embryo.

## MATERIALS AND METHODS

### Animal experiments

Animal experiments were approved by, and conducted according to ethics guidelines from the Shanghai Institute of Planned Parenthood Research (China), Wenzhou Medical University (China) and the Kolling Institute, University of Sydney (Australia). FVB mice and heterozygous *Eif4e*^+/−^ mice were obtained from the Institute of Developmental Biology and Molecular Medicine of Fudan University (Shanghai, China). *Eif4e*^+/−^ mice were generated by random germline transposition of *PB [Act-RFP]* (Table S2), a PB transposon, into the FVB/N background (Ding et al., 2005). Hybrid (C57BL/6×CBA/He) mice in some experiments were housed and bred in the Gore Hill Research Laboratory (St Leonards, Australia). Experiments were performed at the Shanghai Institute of Planned Parenthood Research (China), Wenzhou Medical University (China) and the Kolling Institute, University of Sydney (Australia).

### Embryo collection and culture

Six-week-old females were superovulated by intraperitoneal injection of 5 IU pregnant mare serum gonadotropin (PMSG) (Ningbo Second Hormone Factory). After 48 h, mice were injected again with 5 IU hCG (Livzon). Pregnancy was confirmed by the presence of a copulation plug the following morning. Oocytes or zygotes were recovered 20 h post-hCG from unmated and mated females, respectively. Cumulus cells were removed by brief exposure to 300 IU hyaluronidase (Sigma-Aldrich). Two-cell, four-cell, eight-cell embryos and blastocysts were isolated from the oviducts and/or uterus of plug-positive female mice at 40, 60, 68 and 90 h after hCG injection, respectively. Oocytes and embryos were collected in HEPES-buffered modified human tubal fluid medium (HEPES-HTF; Nanjin Your Bio-tech Development) (O'Neill, 1997). Embryos were cultured at a density of ten embryos in 10 µl KSOM medium (Lawitts and Biggers, 1993), according to the experimental design, in Nunc 60-well plates (LUX 5260; Nunc) overlaid with 2 mm of heavy paraffin oil (Sigma-Aldrich) at 37°C in 5% CO_2_ in air tension. All components of the media were tissue-culture grade (Sigma-Aldrich) and contained 3 mg bovine serum albumin/ml (Sigma-Aldrich). Pharmacological treatments were performed in KSOM medium supplemented with: (1) 1, 10, 100 or 1000 nM of 2-(4-amino-1-isopropyl-1H-pyrazolo[3,4-d]pyrimidin-3-yl)-1H-indol-5-ol (PP242; Sigma-Aldrich); (2) 2, 10 or 50 µM 4EGI-1 (Merck); (3) 10 µM SB203580 (Merck) (Jin and O'Neill, 2014; Sekiyama et al., 2015); (4) 25 nM staurosporine (Merck) (Jin and O'Neill, 2014); (5) 37.5 µM OPP (Thermo Fisher Scientific) or 0.1, 1 or 10 µM cycloheximide (MedChemExpress).

### Click-iT^®^ Plus OPP Alexa Fluor^®^ protein synthesis assay

Changes in protein expression were analyzed using Click-iT^®^ Plus OPP Protein Synthesis Assay Kits (Thermo Fisher Scientific). The procedures were modified based on the manufacturer's instructions. In brief, the one-cell or two-cell embryos from wild-type mating or *Eif4e*^+/−^ cross-mating females were treated in OPP in KSOM medium without amino acids for several time periods depending on the experimental design. The treated embryos were fixed and permeabilized followed by Click-iT Plus OPP Detection and DNA Staining with HCS NuclearMask Blue Stain (Thermo Fisher Scientific). Imaging and analysis were performed with filters appropriate for DAPI/Hoechst, fluorescein isothiocyanate (FITC) for Alexa Fluor^®^ 488 and 560 nM for RFP. Nascent protein synthesis was assessed by the change in signal intensity in the fluorescent channel compared with the control. All quantitative analyses of OPP and RFP fluorescence were performed with the Histogram function within ImageJ (version 1.25). The area of the whole embryo was outlined using the area of interest (AOI). The sum of the intensity of the staining was used for analysis.

### Allelic expression of *Eif4e*

We used a public dataset generated by Deng et al. (2014) to assess the relative contribution of transcription from the maternally or paternally derived alleles to the transcripts present within embryos at each stage of preimplantation development. This dataset used the global analyses of allelic expression across individual cells of mouse preimplantation embryos of mixed background [CAST/EiJ (maternal)×C57BL/6J (paternal)]. Reads that occurred across informative single-nucleotide polymorphisms (SNPs) could be allocated as being derived from one or other of the parental alleles (Table S3).

### Monitoring of RFP expression

*In vitro* embryos expressing the RFP transgene were monitored with an inverted epifluorescent microscope (TS-2R, Nikon) at 24 h intervals. Baseline fluorescence was set as the measured fluorescence in wild-type embryos.

### Genotyping

Genomic DNA was extracted from mouse tails and individual embryos with either 180 μl or 5 μl 50 mM NaOH at 95°C for 10 min, followed by either 20 μl or 0.5 μl 1 M Tris-HCl (pH 8.0), respectively. DNA was used as templates for PCR (KOD FX, TOYOBO) with the primer pairs: *PB* primer (RF1) CCTCGATATACAGACCGATAAAACACATGC, GL primer TGCTTATCAACAAAAAGCAGATGGC, GR primer ACAGGAAAGGAGACAGTACCTGAG. The insertion band size was 580 bp and GL/GR was 949 bp. PCR amplification products were analyzed by electrophoresis on 2% (w/v) agarose gel staining with SYBR green to visualize PCR products on an ultraviolet (UV) transilluminator (Thermo Fisher Scientific). Fragments were verified by size and the sequences of representative samples were analyzed and compared with mouse *Eif4e* by BLAST search (Shanghai Jieli Science and Technology).

### IVF and ICSI

Concentrated sperm were collected from the epididymis of 10- to 12-week-old hybrid males and incubated in pre-equilibrated HTF (Quinn et al., 1985) for 15-30 min at 37°C with 5% CO_2_. Cumulus-oocyte complexes (COCs) were collected from oviducts of female hybrids 13-15 h post-hCG and briefly treated with hyaluronidase HEPES-HTF medium. COCs were washed and moved into HTF medium in preparation for ICSI.

For IVF, sperm were observed under an Olympus IX75 microscope. Fresh sperm were incubated in drops of equilibrated HTF for 1 h at a final concentration of 1×10^6^/ml. COCs were added and incubated for 6 h. Fertilized oocytes at the pronuclear stage were picked out and transferred to 20 µl KSOM medium drops (20 embryos per drop).

For ICSI, 1 μl of fresh sperm was mixed with a drop of HEPES-HTF medium containing 10% (w/v) polyvinylpyrrolidone (PVP, Sigma-Aldrich). Piezo pulses were used to separate the sperm head from the tail so that the head could be injected into the oocyte. The embryos were observed for pronuclear embryos, two-cell embryos and blastocysts at 6 h, 24 h and 96 h after insemination, respectively.

### Blastocyst outgrowth

Blastocysts developed *in vitro* were cultured in each well of a 24-well plate with 1 ml Dulbecco's modified Eagle medium (DMEM, Sigma-Aldrich) supplemented with 10% (v/v) heat-inactivated (40 min at 56°C) fetal bovine serum (FBS, Sigma-Aldrich), 0.6 mg penicillin/ml and 1 mg streptomycin/ml (Sigma-Aldrich). After 96 h, the embryos were analyzed with genotyping and immunofluorescence.

### Immunofluorescence

Immunofluorescence was performed as previously described (Jin and O'Neill, 2010, 2014). After fixation, permeabilization and blocking, embryos or sperm were incubated overnight at 4°C with primary antibodies: 2 μg/ml rabbit anti-eIF4E polyclonal IgG (ab1126, Abcam), 2 μg/ml rabbit anti-p-eIF4E (S209) polyclonal IgG (ab76256, Abcam), 2 μg/ml mouse anti-4E-BP1 polyclonal IgG (ab47719, Abcam), 2 μg/ml rabbit anti-p4E-BP1+2+3 (T45) (EPR2169Y) polyclonal IgG (ab68187, Abcam), 2 μg/ml rabbit anti-OCT3/4 polyclonal IgG (ab18976, Abcam), 2 μg/ml rabbit anti-p-S6K1 (T389) polyclonal IgG (ab2571, Abcam), anti-mouse mTOR monoclonal IgG (ab87540, Abcam), anti-rabbit p-mTOR (ser2448) polyclonal IgG (ab109268, Abcam) and 2 μg/ml isotype negative-control rabbit IgG or mouse IgG (Sigma-Aldrich) according to the animal source for primary antibodies. Primary antibodies were detected by 1:150 Texas Red-conjugated goat-anti mouse (Sigma-Aldrich) or 1:150 FITC-conjugated goat anti-rabbit (Sigma-Aldrich) secondary antibodies for 1 h at room temperature. Whole-section imaging was performed by mercury lamp UV illumination with an epifluorescent Nikon ECLIPSE 80i microscope, using a Plan Apo 40×/1.0 oil objective. Optical sectioning was performed with a Nikon A1+ confocal microscope equipped with a Plan Apo 60× oil objective. All quantitative analysis of immunofluorescence experiments was performed with the Histogram function within Image-Pro Plus (version 6.3, Media Cybernetics). The area of the epiblast or whole embryo was outlined using the AOI. The sum of the intensity of the staining by the primary antibody of interest was used for analysis.

### Western blot

Western blot analysis was performed as previously described (Jin and O'Neill, 2010). The embryos were washed in ice-cold PBS, lysed in extraction buffer, followed by three cycles of freezing and thawing in liquid nitrogen and by vortexing, respectively. The extracted embryo proteins were diluted with Laemmli loading buffer (Bio-Rad), separated on 20% homogenous SDS-polyacrylamide gels (GE Healthcare Australia) using a PhastSystem workstation (Amersham Pharmacia Biotech). Proteins were then transferred onto polyvinylidene fluoride membrane (PVDF, Hybond-P, Amersham) in transfer buffer containing 12 mM Tris (Sigma-Aldrich), 96 mM glycine (BDH) and 20% (v/v) methanol (BDH) by a semi-dry PhastTransfer system (Amersham). Western blot analysis of RFP, mTOR and p-mTOR was performed on a Bio-Rad system, according to the manufacturer's instructions. In brief, 30 µl of a sample containing denatured tissue protein or embryo protein and loading buffer was loaded on Mini-PROTEAN^®^ TGX™ Precast Gels (Bio-Rad) for separation. Proteins were transferred onto a PVDF membrane with a mixed-molecular-weight setting using the Trans-Blot Turbo Transfer System (Bio-Rad).

The membrane was incubated overnight at 4°C in 10 ml of blocking buffer containing 2.5% (w/v) skim milk powder (Diploma) with 0.2 μg/ml primary antibodies. The membrane was then incubated in 1:5000 horseradish peroxidase (HRP)-conjugated secondary antibody (Sigma-Aldrich) followed by SuperSignal West Femto Maximum Sensitivity Substrate (Thermo Fisher Scientific) and the products were recovered using the VersaDoc MP 4000 system (Bio-Rad). To detect other antigens on the same samples, the membrane was either directly reprobed or stripped by incubation in 200 mM NaOH (Sigma-Aldrich) for 30 min at room temperature and then reprobed with other primary antibodies. Loading controls were used to detect housekeeping genes with 1:2000 rabbit anti-actin IgG (A2066, Sigma-Aldrich) or 1:1000 rabbit anti-beta tubulin HRP-conjugated IgG (ab21058, Abcam). The bands were analyzed quantitatively with LabWorks Image Acquisition and Analysis Software Ver 4.5 (UVP). The integrated optical density (IOD) of each band was measured. The relative IOD was the ratio of the IOD of the targeted band compared with the IOD of the loading control. The molecular weights of the bands were determined with LabWorks Image Acquisition and Analysis Software by comparing them with the loaded prestained protein ladder (Precision Plus Protein Dual Xtra Prestained Protein Standard 1610377, Bio-Rad).

### Statistical analysis

Statistical analysis was performed with SPSS for Windows (version 22.0). Fluorescence intensity (AU, arbitrary units of optical density of staining), IOD, area and cell number were analyzed quantitatively by univariate analysis of variance. Those parameters were set as the dependent variables, whereas the test treatments, inhibitor doses, developmental stages and genotypes were the independent variables. Experimental replicates were incorporated into the model as covariates. Differences between individual independent variables were analyzed by the least significance difference test. Blastocyst development rate was assessed by binary logistic regression analysis. Chi-squared tests were used to determine the differences between observed and expected frequency distributions. *P*<0.05 was considered significant.

## Supplementary Material

Supplementary information

Reviewer comments
