# Supplementary material for: Regulation of the mammalian maternal-to-embryonic transition by eukaryotic translation initiation factor 4E
Source: Development. 2021 Jun 25;148(12):dev190793. doi: 10.1242/dev.190793 (PMC8254863; doi:10.1242/dev.190793)
Supplement: Supplementary information [file develop-148-190793-s1.pdf]

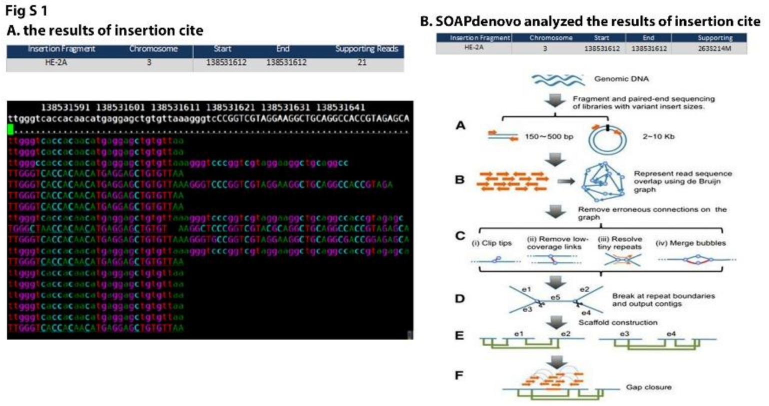

Fig. S1. Analysis of *PB[Act-RFP]* insertion by sequencing of *Eif4e$^{+/-}$* mouse genome

DNA was extracted from transgene mouse (*Eif4e$^{+/-}$)* peripheral blood. its concentration was detected using Qubit™ Flex Fluorometer and genome integrity was detected using agarose gel electrophoresis. Qualified DNA were submitted to BGI (Shenzhen, China) for library preparation and sequencing on BGISEQ-500 platform. A total 211G clean data was obtained with a 75X average depth. BWA (version 0.7.17-r1188) with MEM was used to align sequence reads, clone sequences and assembly contigs to reference mm38 to create sequence alignment/map (SAM) format, giving rise to an average 99.75% mapping ratio. Samtools was used to select high quality mapped reads with mapQ value >30 for insertion site analysis.

After alignment of soft clip reads and pair end reads to reference genome and compare with average library size, the aligned sites of soft clip reads with the supporting number greater than 2 were recognized to be insertion sites. Only one insertion cite was found on Chromosome 3, 138531612 **(A)**. Furthermore, with SOAPdenovo reads were assembled to find the sites where insertion was assembled. The results also showed an unique cite of insertion on Chromosome 3, 138531612 (**B**).

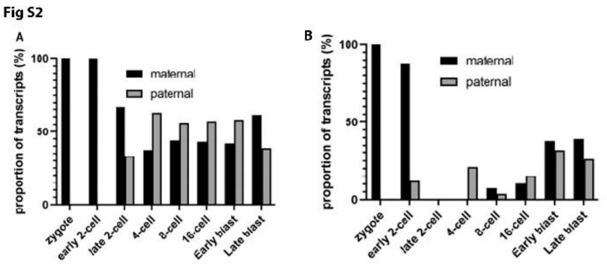

Fig. S2. Allelic source of *Eif4e* transcripts across each stage of development of the mouse embryo.

(A) The proportion (%) of all reads that could be assigned to one or other of the parental alleles. Source data is in supplementary DATA 1. **(B)** The proportion of individual cells (%) at each stage of where detected transcripts came exclusively from one or other of the parental alleles.

Table S1. The structure of PB[Act-RFP]

Click here to download Table S1

Table S2. The number of embryos and RFP+ embryos in developmental landmarks

	No. of Mating female	No. of virginal plug	Zygotes			2-cell embryo			4-8 cell embryo		
			Mean ± S.E.M	total		Mean ± S.E.M	total		Mean ± S.E.M	total	
				No	RFP+		No	RFP+		No	RFP+
Eif4e$^{+/-}$ ♂ × ***Eif4e$^{+/-}$*** ♀	90	37 (31.1%)*	15.4 ± 1.1	202	96 (47.5%)	12.7 ± 0.8**	151	0 (0%)	11.9 ± 1.4*	64	44 (68.8%)
Eif4e$^{+/+}$ ♂ × ***Eif4e$^{+/-}$*** ♀	25	23 (92.0%)	16.3 ± 0.8	147	72 (49%)	15.8 ± 1.1	110	58 (52.3%)	16.3 ± 0.9	134	65 (48.5%)
Eif4e$^{+/-}$ ♂ × ***Eif4e$^{+/+}$*** ♀	29	25 (86.2%)	16.2 ± 1.1	80	0 (0%)	16.1 ± 0.9	81	0 (0%)	16.1 ± 1.3	90	72 (40.0%)
Eif4e$^{+/+}$ ♂ × ***Eif4e$^{+/+}$*** ♀	20	18 (90%)	20.2 ± 0.8	165	NA	18.3 ± 0.6	183	NA	NA		

Discovering plugs in female virginals indicate fertilization. Its rate, the number of embryos and RFP^{+} embryos were shown in several developmental stages that freshly collected from female reproductive tracts. *p<0.001 and **p <0.01, compared to the according to *Eif4e$^{+/+}$*♂ × *Eif4e$^{+/+}$*♀ mating or *Eif4e$^{+/-}$*♂ × *Eif4e$^{+/+}$*♀ mating *and Eif4e$^{+/+}$*♂ × *Eif4e$^{+/-}$*♀.

Development • Supplementary information

Table S3. Allelic source of Eif4e transcripts across each stage of development of the mouse embryo

Click here to download Table S3
