# Supplementary material for: Regulation of the mammalian maternal-to-embryonic transition by eukaryotic translation initiation factor 4E
Source: Development. 2021 Jun 25;148(12):dev190793. doi: 10.1242/dev.190793 (PMC8254863; doi:10.1242/dev.190793)
Supplement: Reviewer comments [file develop-148-190793_review_history.pdf]

Regulation of the mammalian maternal-to-embryonic transition by eukaryotic translation initiation factor 4E

Yan Li, Jianan Tang, Xu Ji, Min-Min Hua, Miao Liu, Lu Chang, Yihua Gu, Changgen Shi, Wuhua Ni, Jing Liu, Hui-juan Shi, Xuefeng Huang, Christopher O'Neill and Xingliang Jin
DOI: 10.1242/dev.190793

Editor: Patrick Tam

Review timeline

Original submission:	29 March 2020
Editorial decision:	12 May 2020
First revision received:	25 March 2021
Editorial decision:	16 April 2021
Second revision received:	10 May 2021
Accepted:	12 May 2021

Original submission

First decision letter

MS ID#: DEVELOP/2020/190793

MS TITLE: The regulation of the mammalian maternal-to-embryonic transition by Eukaryotic translation initiation factor 4E

AUTHORS: Yan Li, Xuefeng Huang, Jianan Tang, XU JI, Miao Liu, Lu Chang, Jing Liu, Yihua Gu, Changgen Shi, Wuhua Ni, Hui-juan Shi, Chris O'Neill, and Xingliang Jin

I have now received all the referees' reports on the above manuscript, and have reached a decision. The referees' comments are appended below, or you can access them online: please go to BenchPress and click on the 'Manuscripts with Decisions' queue in the Author Area.

As you will see, the referees express considerable interest in your work, but have some significant criticisms and recommend a substantial revision of your manuscript before we can consider publication. If you are able to revise the manuscript along the lines suggested, which may involve further experiments, I will be happy receive a revised version of the manuscript. Your revised paper will be re-reviewed by one or more of the original referees, and acceptance of your manuscript will depend on your addressing satisfactorily the reviewers' major concerns. Please also note that Development will normally permit only ONE round of major revision.

We are aware that you may currently be unable to access the lab to undertake experimental revisions. If it would be helpful, we encourage you to contact us to discuss your revision in greater detail. Please send us a point-by-point response indicating where you are able to address concerns raised (either experimentally or by changes to the text) and where you will not be able to do so within the normal timeframe of a revision. We will then provide further guidance. Please also note that we are happy to extend revision timeframes as necessary.

Please attend to all of the reviewers' comments and ensure that you clearly highlight all changes made in the revised manuscript. Please avoid using 'Tracked changes' in Word files as these are lost in PDF conversion. I should be grateful if you would also provide a point-by-point response detailing

© 2021. Published by The Company of Biologists under the terms of the Creative Commons Attribution License (https://creativecommons.org/licenses/by/4.0/).

how you have dealt with the points raised by the reviewers in the 'Response to Reviewers' box. If you do not agree with any of their criticisms or suggestions please explain clearly why this is so.

Reviewer 1

Advance summary and potential significance to field

This study offers original insights into the oocyte-to-embryo transition as taking place at the protein level of gene expression, in mice. This level has generally been underrated so far, and the present study is timely.
Study proposes that EIF4E (factor essential for the initiation phase of translation) is a new recruit to the family of critical maternal products that are stored in the oocyte and that guide the transition, since EIF4E -/- embryos form defective blastocysts (deficit in pluripotent ICM); study also suggests the mechanistic regulation EIF4E may be involved in (PI3kinase/AKT/mTOR signaling pathway).

Comments for the author

Background
The protein / translational dimension of the oocyte to embryo transition lags behind, compared to the mRNA-based characterization of mammalian embryogenesis. Some catching-up is overdue. The Authors dissected the requirement of EIF4E for mouse pre- and peri-implantation development. EIF4E is the cap-binding protein and therefore a key element of the process of mRNA translation into protein, particularly the initiation thereof.
It has been known that while Eif4e heterozygous mutant mice are viable, the homozygous mutants die shortly after implantation (confirmed in this study), for unclear reasons. Therefore, the Authors set out to illuminate the bases of this lethality, determining, for example, when exactly the Eif4e -/- concepti would die, and how.
To this end, the Authors performed a knockout of Eif4e, by intercross of heterozygotic parents which were generated via a method of insertional mutagenesis mediated by PiggyBac (PB) [Act-RFP] transposition. The Authors rely on expression of RFP as a means of genotypization. RFP negative embryos are wt, RFP positive embryos are either +/- or -/- (the latter fluoresce brighter). The results of the study give strong support to the conclusion that EIF4E is a critical maternal product, necessary to form pluripotent blastocysts, and that EIF4E is regulated through the PI3kinase/AKT/mTOR signaling pathway.

Major remarks
1.
Can the Authors please provide more information about the insertional mutagenesis in this specific case? I understand that the insertion was localized in the Eif4e locus; but was this the sole insertion in the genome?
Since the PiggyBac transposon targets TTAA sequences, did the Authors verify that only the Eif4e locus was mutated? It would be helpful (for the readership) to spend a sentence or two about this basic characterization, in the Materials and Methods.
2.
Given the insertion, which is present in one allele but not in the other (parental animals), I wonder if a better name to describe the genotype would be ‘hemizygous’ instead of ‘heterozygous’ (in the following I will keep the Authors’ nomenclature i.e. heterozygous, even though I’m not sure). If you see my point, then please change the name, otherwise leave it as it is.
3.
Can the Authors please provide more information about the nature of the Act-RFP? This product exists on its own, not as a fusion with EIF4E, correct? If RFP is a reporter, as the Authors name it, then it assays for the activity of a particular promoter (EIF4E) and it is expressed as a separate molecule. If so, then why does Act-RFP reflect the expression profile of the phosphorylated EIF4E so closely, disappearing abruptly at the 2-cell stage, and then reappearing at the 4-cell stage (compare Figure 1D and 2D)? I’m having difficulties at understanding why the oocyte would ‘bother’ to degrade Act-RFP so effectively, unless Act-RFP were fused to EIF4E. Please comment on this (in the rebuttal letter will be fine).
4.

© 2021. Published by The Company of Biologists under the terms of the Creative Commons Attribution License (https://creativecommons.org/licenses/by/4.0/).

Further to Act-RFP: the RFP signal is present in all MII oocytes (supposedly from heterozygotic females) but it disappears in half of the 1-cells/zygotes that were fertilized by sperm regardless if the spermatozoa were from +/- (Fig.2D) or +/+ (Fig. 4B) males. Therefore, the 50% observed at the 1-cell stage has probably to do with the oocyte rather than with sperm. I'm puzzled by the 50% and its kinetics, because all of the oocytes of +/- females accumulated RFP during oogenesis. One possibility is that the RFP went degraded after oocyte activation, only in those oocytes that had lost the mutated allele to the polar body; another possibility is that the RFP went degraded to a similar extent in all of the activated oocytes, but was replenished only in those that retained the mutated allele in the ooplasm (not in the polar body). I think this 50% of RFP-negative zygotes presupposes a very high rate of either degradation or synthesis, considering the short window of time we are looking at (few hours into the first cell cycle). I wish the Authors elaborated more on this (text, no experiments required).
5.
The Authors state that “Maternal stores of eIF4E supported development up to the 2-4-cell stage after which new expression occurred from both alleles.”. I personally accept the statement, however I also think that the conclusion could benefit from e.g. single nucleotide polymorphism analysis or allele-specific expression analysis of the transcripts. There are several datasets that can be consulted e.g. PMID 24408435 (in this dataset I can see eIF4E). No new experiments are needed. Granted, PMID 24408435 does not feature a protein analysis, but the mRNA could provide the Authors with additional support for the view that the paternal source joins the game late in preimplantation development, and therefore the mutant phenotype primarily depends on the maternal source. How? If one saw that in 2-cell and 4-cell embryos the eIF4E mRNA is mainly expressed from the maternal part of the embryonic genome.
6.
The deficit of Eif4e in the bright RFP-positive embryos is documented in Figure 3C and 3D. I note that there is still some (approx. 15%) of the EIF4E protein left at the late blastocyst stage. Parenthetically, I think that the EIF4E protein quantification in -/- embryos should be documented also by Western blot analysis, if the required numbers of cells permit (the legend to Figure 1 mentions 50 oocytes or embryos per lane). Had EIF4E been removed completely, could the phenotype have been different e.g. more severe? The data of the pharmacological inhibition (Figure 2 E,F) suggest a block at the 2-4 cell stage. However, the inhibitor does not distinguish between maternal (oocytic) and embryonic (de novo synthesized, including paternal) source of EIF4E, and it inhibits both. If the sole maternal deposit of EIF4E was tackled (along the lines of PMID 31638890, PMID 31777931), would the Authors expect to observe a similar phenotype to the one they observed after applying the pharmacological inhibitor? Please answer in text, no experiments requested.
7.
EIF4E can be regarded as serving a housekeeping function of the cell. Disruption of this locus can impact on any cellular process, directly or indirectly. Therefore, I think the effect of the EIF4E knockout should also be measured on the global (genome-wide) scale, in addition to the valid (but partial) characterization presented in this study. Does global protein synthesis suffer? (HPG or OPP assays, see below). How does the global gene expression profile change? (see below). I think experimental answers are needed here. Given the housekeeping function of EIF4E, it is difficult for me to imagine that the only problem of -/- embryos lies in their ICM expressing little Oct4 - although Oct4 certainly is part of the problem. Looking at Figure 3A I'd say that also the trophectoderm could have a problem (reduced ability to expand and attach, which speaks for other genes e.g. TEAD4, CDX2).

Minor remarks
1.
A few typos / syntax errors that need fixed:
Page 5: sumulation Page 5: in the in the Page 14: it's presence at the critical embryonic transitions Page 18: 20% homogenous SDS-polyacrylamide gels Page 25: propidium iodine
2.
Please specify if the KSOM medium used for in vitro embryo culture contained aminoacids, or not. Aminoacids are key for outgrowth formation, and can influence the results substantially.
3.
Could the Authors demonstrate that there is, as one would expect, less global translation in the -/- embryos?

© 2021. Published by The Company of Biologists under the terms of the Creative Commons Attribution License (https://creativecommons.org/licenses/by/4.0/).

I've read the sentence: "Furthermore, Eif4e +/- cells showed no apparent change in global protein synthesis (12, 32)" in Discussion. In my opinion the Authors should not rely on other studies, but examine their own biologic material i.e. test -/- embryos at the 2 or 4 cell stage, for protein synthesis. As a positive control, a reduction of protein synthesis should be observed in the presence of the pharmacological inhibitor of eIF4E 4EGI-1. Possible ways to find out would be using the L-homopropargylglycine (HPG) or the O-propargyl-puromycin (OPP) assay, for example.
4.
Suggestion (take it or leave it). I encourage the Authors to think about a way to 'simplify' the biological system, because while it is very likely that the paternal allele is not the key player in the phenotype being studied here, it is also true that there is some kind of maternal-paternal genomic interaction, which we don't fully understand yet (see Table 6). My suggestion would be to consider a parthenogenetic activation of the +/- oocytes, so as to be able to focus on the maternal source without the confounder of sperm contribution.

Conclusions
I think the biological problem addressed in the study is an important one, and the Authors produced a very detailed set of data that recruit EIF4E to the family of maternal factors important for the oocyte-to-embryo transition, in mice. My only 'problems' are that EIF4E is still there (albeit reduced by a substantial extent) and that less Oct4 is unlikely to be the only deficit in the -/- blastocysts. So, my question is: did the residual amount postpone a phenotype which would have occurred earlier, and perhaps even more severely, had the maternal source been depleted to a larger extent? It is probably unnecessary and also biologically impossible (my opinion) to remove a gene product down to the last molecule. Very stable proteins can hang around in the embryo even if the locus was excised conditionally during oogenesis. Therefore, the inactivation / inhibition of a protein of interest may be sufficient. To this end, the Authors used pharmacological inhibition but as I noted before, the method does not distinguish between maternal (oocytic) and embryonic (paternal) gene product. I think the Authors should acknowledge this limitation and elaborate on it, in writing. Last, I note that if the Authors ascribe EIF4E with a key role in the oocyte-to- embryo transition, then they invite the question: what is the impact genome-wide of disrupting EIF4E, in terms of e.g. downstream transcriptome?
When Brg1 was eliminated by Bultman et al. using a conditional KO strategy, these Authors observed a major perturbation of embryonic gene expression (30% of the genes were affected). It is difficult for me not to think that if global effects be expected of the mutation (=translation process), then also the assay should be global. While I think that the conclusions of this study won't change, adding this in formation would make the study complete.
I hope you find these comments useful.

Regards,
Michele Boiani

Reviewer 2

Advance summary and potential significance to field

Li et al. show that the lack of eIF4E results in pri-implantation embryonic lethality and eIF4E hypomorphic mice exhibit a growth defect. They also demonstrate that maternal eIF4E largely contributes to development of 2-4-cell embryo. Pharmacological treatments were employed to modulate the activity of eIF4E and study its impact on early embryogenesis. This study addresses an important question

Comments for the author

In this manuscript Li et al. examined the role of the cap-binding protein eIF4E in early embryogenesis by generating a PB [Act-RFP] transposon eIF4E knockout mouse. Although the importance of eIF4E in development has been previously established, a detailed analysis of its functional importance and its contribution to EMT has not been addressed. This study addresses an important question. However, I have major concerns about the data that detract from the significance of the conclusions (see below).
Major comment:

© 2021. Published by The Company of Biologists under the terms of the Creative Commons Attribution License (https://creativecommons.org/licenses/by/4.0/).

1. The study lacks any genuine translation experiments, while focusing on a key translation initiation factor. Examining the global changes in translation (e.g. using Op-puro, metabolic labeling) or examining some of the well-established eIF4E targets is lacking.

2. Page 11: The authors state: “The phosphorylated form (pT45 4E-BP1+2+3) was also widely distributed “

This argument is puzzling concerning the literature about 4E-BP phosphorylation sites. The mTORC1 phosphorylates 4E-BPs at T37/46. The source of pT45 4E-BP1+2+3 antibody has not been indicated in the manuscript.

3. Fig 5. Blocking the phosphorylation of 4E-BP1 and S6K1 by 4EGI-1 in Fig. 5 is unprecedented and without some validation and supporting experiments, it raises concerns about the validity of the results and conclusion.

Minor comments:
4. Page 5: a post-translational modification, such as sumulation should be changed to “sumoylation”

5. Figure 1A. Must add the ACTIN blot to the figure.

6. Figure 1B. Contrary to the authors’ claim immunofluorescence images show that the distribution of eIF4E (midpiece) is different from p-eIF4E (head).

7. Fig 2D, Table 2, suppl table1 showed that “No morphological 2-cell stage embryos collected from the reproductive tract were RFP+”. However, in Figure 1D there is an abundant expression of eIF4E in 2-cell stage and Figure 4B shows the expression of RFP at two-cell stage.

8. WB in Figure 4C is inconsistent with 1D.

9. It is puzzling that the high rate of embryonic loss by the 2-cell stage has no impact on the blastocyst stage ($p > 0.05$).

10. Page 10: The meaning of the sentence is not clear: “While phosphorylation has some control functions, a dominant regulator of eIF4E is eukaryotic translation initiation factor 4E-binding protein 1 (4E-BP1)”.

First revision

<u>Author response to reviewers' comments</u>

Reviewer 1
Major remarks

1.

Can the Authors please provide more information about the insertional mutagenesis in this specific case? I understand that the insertion was localized in the Eif4e locus; but was this the sole insertion in the genome? Since the PiggyBac transposon targets TTAA sequences, did the Authors verify that only the Eif4e locus was mutated? It would be helpful (for the readership) to spend a sentence or two about this basic characterization, in the Materials and Methods.

A: We have now added a more detailed section describing the sequencing approach (Page 5, Lines 137- 139) that confirmed that the insert occurs in only one location and this is within intron 2 of the eIF4E locus. This detail in now within the results, materials and methods (page5 and line 137-138s) and a new Fig S1. We believe this extra data addresses the

© 2021. Published by The Company of Biologists under the terms of the Creative Commons Attribution License (https://creativecommons.org/licenses/by/4.0/).

referee's question and confirms the validity of the model.
2.

Given the insertion, which is present in one allele but not in the other (parental animals), I wonder if a better name to describe the genotype would be 'hemizygous' instead of 'heterozygous' (in the following I will keep the Authors' nomenclature i.e. heterozygous, even though I'm not sure). If you see my point, then please change the name, otherwise leave it as it is.

A: eIF4E is located on autosomal Chromosome 3. Our understanding is that the term hemizygous is most commonly used in conjunction with the sex chromosomes and sex-linked genes. Although it could be argued that the insertion of a gene (RFP) creates a hemizygous state we feel that the use of heterozygous is a more commonly used and understood terminology with transgenic models as used here. We think that the continued use of this terminology is less likely to create confusion with non- specialist readers.

3.

Can the Authors please provide more information about the nature of the Act-RFP? This product exists on its own, not as a fusion with EIF4E, correct? If RFP is a reporter, as the Authors name it, then it assays for the activity of a particular promoter (EIF4E) and it is expressed as a separate molecule. If so, then why does Act-RFP reflect the expression profile of the phosphorylated EIF4E so closely, disappearing abruptly at the 2-cell stage, and then reappearing at the 4-cell stage (compare Figure 1D and 2D)? I'm having difficulties at understanding why the oocyte would 'bother' to degrade Act-RFP so effectively, unless Act-RFP were fused to EIF4E. Please comment on this (in the rebuttal letter will be fine).

A: We have now added more detail of the construct in the suppl File 1. We have also added further data to the text showing Western blot analysis of RFP across a range of tissues in transgenic mice (Fig 2 B) (Page 5, Line 139-141). This shows that the signal is of the expected size for RFP. A result that is not consistent with it being a fusion protein, although we have not formally tested this. The levels detected showed large differences between tissues suggesting active regulation of its expression. While we expect that its expression occurs as a consequence of the combined promoter activities of eIF4e and ACT we have not formally proven this and have therefore removed claims of it being a direct reporter of eIF4e gene expression activity.
The active degradation of most maternally inherited proteins via ubiquitin-mediated pathways is a well- known characteristic of the newly fertilized embryo. We expect that the inherited RFP is also subject to this degradation, and this accounts for the characteristic patterns of expression noted by the reviewer. A noteworthy observation was that the rate of loss of RFP seemed to differ depending upon whether embryos were collected directly from the reproductive tract or cultured in vitro (degradation was slower in cultured embryos). This difference did complicate the analysis between the models used and may account for some nuanced differences in outcomes. This difference was not formally investigated as part of this study and has therefore not been canvassed within the manuscript. The information is added here to provide the reviewers with some insight into the idiosyncrasies of the model. We intend to more fully investigate and report these apparent effects of culture on the half-life of RFP in the early embryo in the near future.

4.

Further to Act-RFP: the RFP signal is present in all MII oocytes (supposedly from heterozygotic females) but it disappears in half of the 1-cells/zygotes that were fertilized by sperm regardless if the spermatozoa were from +/- (Fig.2D) or +/+ (Fig. 4B) males. Therefore, the 50% observed at the 1-cell stage has probably to do with the oocyte rather than with sperm. I'm puzzled by the 50% and its kinetics, because all of the oocytes of +/- females accumulated RFP during oogenesis. One possibility is that the RFP went degraded after oocyte activation, only in those oocytes that had lost the mutated allele to the polar body; another possibility is that the RFP went degraded to a similar extent in all of the activated oocytes, but was replenished only in those that retained the

© 2021. Published by The Company of Biologists under the terms of the Creative Commons Attribution License (https://creativecommons.org/licenses/by/4.0/).

mutated allele in the ooplasm (not in the polar body). I think this 50% of RFP-negative zygotes presupposes a very high rate of either degradation or synthesis, considering the short window of time we are looking at (few hours into the first cell cycle). I wish the Authors elaborated more on this (text, no experiments required).

A. We have added some commentary on this helpful comment into the revised manuscript (Page 6 and line171-179)

5.

The Authors state that "Maternal stores of eIF4E supported development up to the 2-4-cell stage after which new expression occurred from both alleles.". I personally accept the statement; however, I also think that the conclusion could benefit from e.g. single nucleotide polymorphism analysis or allele- specific expression analysis of the transcripts. There are several datasets that can be consulted e.g. PMID 24408435 (in this dataset I can see eIF4E). No new experiments are needed. Granted, PMID 24408435 does not feature a protein analysis, but the mRNA could provide the Authors with additional support for the view that the paternal source joins the game late in preimplantation development, and therefore the mutant phenotype primarily depends on the maternal source. How? If one saw that in 2-cell and 4-cell embryos the eIF4E mRNA is mainly expressed from the maternal part of the embryonic genome.

A. Thank you for this helpful suggestion. We have now undertaken this analysis and include new figures (Fig S2) and Suppl File 3, showing this analysis of the parental origins of allelic expression of Eif4e (Text on Page 8, Line 209-220).

6.

The deficit of Eif4e in the bright RFP-positive embryos is documented in Figure 3C and 3D. I note that there is still some (approx. 15%) of the EIF4E protein left at the late blastocyst stage. Parenthetically, I think that the EIF4E protein quantification in -/- embryos should be documented also by Western blot analysis, if the required numbers of cells permit (the legend to Figure 1 mentions 50 oocytes or embryos per lane). Had EIF4E been removed completely, could the phenotype have been different e.g. more severe? The data of the pharmacological inhibition (Figure 2 E,F) suggest a block at the 2-4 cell stage. However, the inhibitor does not distinguish between maternal (oocytic) and embryonic (de novo synthesized, including paternal) source of EIF4E, and it inhibits both. If the sole maternal deposit of EIF4E was tackled (along the lines of PMID 31638890, PMID 31777931), would the Authors expect to observe a similar phenotype to the one they observed after applying the pharmacological inhibitor? Please answer in text, no experiments requested.

A. Using immunostaining analysis, we found eIF4E in Eif4e$^{-/-}$ embryos is present to blastocyst and outgrowth embryos. However, to quantify the amount of eIF4E in Eif4e$^{-/-}$ embryos proved to be a technical challenge. The necessity to both genotype and undertake Western blot analysis on the embryos did not allow us to collect sufficient embryos to have confidence in the results of the Westerns. Given the challenges we have experienced during the Covid period in regenerating sufficient animals to collect large numbers of null-embryos we seek the reviewers consideration on this difficult technical question.

7.

EIF4E can be regarded as serving a housekeeping function of the cell. Disruption of this locus can impact on any cellular process, directly or indirectly. Therefore, I think the effect of the EIF4E knockout should also be measured on the global (genome-wide) scale, in addition to the valid (but partial) characterization presented in this study. Does global protein synthesis suffer? (HPG or OPP assays, see below). How does the global gene expression profile change? (see below). I think experimental answers are needed here.

© 2021. Published by The Company of Biologists under the terms of the Creative Commons Attribution License (https://creativecommons.org/licenses/by/4.0/).

Given the housekeeping function of EIF4E, it is difficult for me to imagine that the only problem of -/- embryos lies in their ICM expressing little Oct4 - although Oct4 certainly is part of the problem. Looking at Figure 3A I'd say that also the trophectoderm could have a problem (reduced ability to expand and attach, which speaks for other genes e.g. TEAD4, CDX2).

A. Yes, we have undertaken extra experiments and demonstrated new protein synthesis in early embryo (new Fig4, Results on Page10-11, Line 263-282). This was blocked by cycloheximide and 4EGI-1. Overall the levels of protein synthesis in embryos from Eif4e$^{+/-}$ x Eif4e$^{+/-}$ cross-mating was generally lower than from wildtype crosses. We were unable to successfully genotype the embryos after the protein synthesis assay and it was therefore not possible to convincingly assess whether there was a Eif4e gene-dosage effect on protein synthesis with embryos. We agree that it is unlikely that a defect on Oct4 expression was the sole effect of Eif4e-deficiency. We include this data to show that the transgene results in generalized failure of normal embryo differentiation and organization. This is likely to be a result of the deficiency in a range of proteins resulting from a constraint in translational activity in the embryo. We have added a comment to the manuscript to clarify this point (Page 11, line 273-282).

Minor remarks

1.

A few typos / syntax errors that need fixed:

Page 5 : sumulation

Page 5: in the in the

Page 14: it's presence at the critical embryonic transitions

Page 18: 20% homogenous SDS-polyacrylamide gels

Page 25: propidium iodine

A. Revised in the text

2.

Please specify if the KSOM medium used for in vitro embryo culture contained aminoacids, or not. Aminoacids are key for outgrowth formation, and can influence the results substantially.

A. The KSOM medium without amino acids was used in all Studies with 4E-GI1 (including IVF and ICSI studies), PP242 and OPP assay to avoid the complex effect of Amino acids. The blastocyst outgrowth studies used DMEM media supplemented with FCS, a media well recognized to support this developmental transition (Materials and Methods -Page 15 Line425 - 430; page 18 lines 484-489)

3.

Could the Authors demonstrate that there is, as one would expect, less global translation in the -/- embryos? I've read the sentence: "Furthermore, Eif4e +/- cells showed no apparent change in global protein synthesis (12, 32)" in Discussion. In my opinion the Authors should not rely on other studies, but examine their own biologic material i.e. test -/- embryos at the 2 or 4 cell stage, for protein synthesis. As a positive control, a reduction of protein synthesis should be observed in the presence of the pharmacological inhibitor of eIF4E, 4EGI-1. Possible ways to find out would be

© 2021. Published by The Company of Biologists under the terms of the Creative Commons Attribution License (https://creativecommons.org/licenses/by/4.0/).

using the L- homopropargylglycine (HPG) or the O-propargyl-puromycin (OPP) assay, for example.

A. Please see the results of the extra experiments reported in new Fig 4. These shows 4EGI-1 did repress the level of protein synthesis within the embryo and also showed that the transgene was associated with reduced translation. Unfortunately, the complexities of the model and constraints on embryo availability prevented a definitive assessment of the extent of transgene-dosage relationship with rates of translation.

4.

Suggestion (take it or leave it). I encourage the Authors to think about a way to 'simplify' the biological system, because while it is very likely that the paternal allele is not the key player in the phenotype being studied here, it is also true that there is some kind of maternal-paternal genomic interaction, which we don't fully understand yet (see Table 6). My suggestion would be to consider a parthenogenetic activation of the +/- oocytes, so as to be able to focus on the maternal source without the confounder of sperm contribution.

A: We agree with this suggestion, and have revised the manuscript by removing the data Fig1-B (Sperm immunofluorescence) and Figure 4 from original version. We are undertaking a more detailed analysi of the roles of Eif4e in the gametes which we hope to report separately in the near future.

Reviewer 2

Major comment:

1. The study lacks any genuine translation experiments, while focusing on a key translation initiation factor. Examining the global changes in translation (e.g. using Op-puro, metabolic labeling) or examining some of the well-established eIF4E targets is lacking.

A. Extra Experiments have been performed and added (Figure 4)

2. Page 11: The authors state: "The phosphorylated form (pT45 4E-BP1+2+3) was also widely distributed ". This argument is puzzling concerning the literature about 4E-BP phosphorylation sites. The mTORC1 phosphorylates 4E-BPs at T37/46. The source of pT45 4E-BP1+2+3 antibody has not been indicated in the manuscript.

A. From Abcom. Anti-eIF4EBP1 + eIF4EBP2 + eIF4EBP3 (phosphor T45) antibody [EPR2169Y] ab68187. Added to the manuscript (pages 18 line 496-497)

3. Fig 5. Blocking the phosphorylation of 4E-BP1 and S6K1 by 4EGI-1 in Fig. 5 is unprecedented and without some validation and supporting experiments, it raises concerns about the validity of the results and conclusion.

A: we agreed with this. It is better do with Western blot: We have difficulty in animal breeding during the Covid-19 pandemic, see comments on reviewer 1 above.
Minor comments:

4. Page 5: a post-translational modification, such as sumulation should be changed to "sumoylation"

A: Revised in text (Page 5 Line 121)

5. Figure 1A. Must add the ACTIN blot to the figure.

A: Added

© 2021. Published by The Company of Biologists under the terms of the Creative Commons Attribution License (https://creativecommons.org/licenses/by/4.0/).

6. Figure 1B. Contrary to the authors' claim immunofluorescence images show that the distribution of eIF4E (midpiece) is different from p-eIF4E (head).

A: eIF4E is also in the sperm head where it is phosphorylated. eIF4E in the midpiece is not phosphorylated. We revised the manuscript by removing all data from male side study as reviewer 1 suggested.

7. Fig 2D, Table 2, suppl table1 showed that "No morphological 2-cell stage embryos collected from the reproductive tract were RFP+". However, in Figure 1D there is an abundant expression of eIF4E in 2-cell stage and Figure 4B shows the expression of RFP at two-cell stage.

A; the sentence: "No morphological 2-cell stage embryos collected from the reproductive tract were RFP+" is described in the result section for Eif4e+/- female mated with Eif4e+/- male.
Yes, Figure 4B shows the expression of RFP at two-cell stage. We found that some embryos cultured form the 1-cell to 2-cell stage retained. We have not had an opportunity to formally investigate this difference, but it may reflect differences in the rate of degradation of maternally inherited proteins during culture. If so this may be an important observation and worthy of further future investigation. We revised the manuscript by removing all data from male side study as reviewer 1 suggested, and these data were also removed.

8. WB in Figure 4C is inconsistent with 1D.

A; Fig 1 D is immunostaining for p-eIF4E, Figure 4C (new version in Fig 5 D) is the presented images (PN2-PN4). We reviewed the original data and adjusted the fluorescence level in PN2 and PN5 in Fig 1D.

9. It is puzzling that the high rate of embryonic loss by the 2-cell stage has no impact on the blastocyst stage ($p > 0.05$).

A. The results indicated the embryos that survive to the 2-cell stage have normal developmental ability to blastocyst stage in vitro. Our study also showed that the embryo in vitro (without maternal environment) in this model had better developmental viability than in vivo (Data not shown in the manuscript) and this will be for further investigation.

10. Page 10: The meaning of the sentence is not clear: "While phosphorylation has some control functions, a dominant regulator of eIF4E is eukaryotic translation initiation factor 4E-binding protein 1 (4E-BP1)".

A. Several references have showed it in cell lines. We revised the sentence to "While phosphorylation has some control functions in somatic cells, a dominant regulator of eIF4E is eukaryotic translation initiation factor 4E-binding protein 1 (4E-BP1). (page11, Line 299-300)

Second decision letter

MS ID#: DEVELOP/2020/190793

MS TITLE: The regulation of the mammalian maternal-to-embryonic transition by Eukaryotic translation initiation factor 4E

AUTHORS: Yan Li, Jianan Tang, Xu JI, Min-Min Hua, Miao Liu, Lu Chang, Yihua Gu, Changgen Shi, Wuhua Ni, Jing Liu, Hui-juan Shi, Xuefeng Huang, Christopher O'Neill, and Xingliang Jin

© 2021. Published by The Company of Biologists under the terms of the Creative Commons Attribution License (https://creativecommons.org/licenses/by/4.0/).

I have now received all the referees reports on the above manuscript, and have reached a decision. The referees' comments are appended below, or you can access them online: please go to BenchPress and click on the 'Manuscripts with Decisions' queue in the Author Area.

The overall evaluation is positive, but there are issues that the reviewers have identified would need to be addressed satisfactorily before we may consider the manuscript for publication in Development. Please attend to all of the reviewers' comments in your revised manuscript and detail them in your point-by-point response. If you do not agree with any of their criticisms or suggestions explain clearly why this is so.

We are aware that you may currently be unable to access the lab to undertake experimental revisions. If it would be helpful, we encourage you to contact us to discuss your revision in greater detail. Please send us a point-by-point response indicating where you are able to address concerns raised (either experimentally or by changes to the text) and where you will not be able to do so within the normal timeframe of a revision. We will then provide further guidance. Please also note that we are happy to extend revision timeframes as necessary.

Reviewer 1

Advance summary and potential significance to field

eIF4E is a new recruit to the family of maternal regulators of the oocyte-to-embryo transition. It also exemplifies that maternal proteins can be very stable and persist throughout cleavage, masking earlier phenotypes which go unnoticed when the classic KO mutant intercrosses (+/- x +/-) are used. Therefore from now on, the role eIF4E must be considered in the study not only of early postimplantation development but also in that of early preimplantation development.

Comments for the author

I do appreciate the substantial efforts the Authors put in this revision, including the reporting of new experiments (e.g. Fig.4). My critiques have been addressed in a satisfactory manner. What I add here are suggestions for refinements.

I understand that by using the PB [Act-RFP] transposon in this study (single insertion on Chr.3), RFP is driven – directly - by an actin promoter which in turn is regulated by the eIF4E promoter, given the specific insertion of the transposon. This was important to clarify. Although I am not fully convinced about ‘heterozygous’ vs. ‘hemizygous’ thing (there is no allelic counterpart of PB [Act-RFP] on the other chromosome, because there is no insertion, so it should be called ‘hemizygous’), I’m fine with the use of ‘heterozygous’, to not create confusion. I appreciate the observation that the regulation of RFP seems to vary depending upon whether same-stage embryos are collected directly from the oviduct or subjected to a prior period of in vitro cultureuntil that stage. I am still puzzled by the observation, in Fig. 2E, that all MII oocytes are RFP positive but just few hours later (pronuclear stage) 50% have lost the signal on their way to become 100% negative at the 2-cell stage. I’ve seen the Authors’ text at line 174 and I am fine with the explanation (I also would not know better). There must be some ‘strategy’ in this RFP behavior, but we do not grasp such ‘strategy’ yet. I had asked to characterize the expression of RFP by isolating the maternal and paternal contribution from each other (wt female x +/- male; +/- female x wt male), and I appreciate Figure S2 (based on Deng et al., 2014) and Table S1. I am also very pleased with the analysis of protein synthesis (Fig.4) and I understand the difficulty of performing the assay of protein synthesis and then also genotypizing the embryos afterwards. All in all, I am pleased with the revision.

I would like to make some final remarks aimed at making the study even more nice and round. Essentially – from my perception - the study shows that unlike the early postimplantation lethality of the classic genetic KO of eIF4E (heterozygotic intercross) (Truitt et al., 2015 and present work with PB [Act-RFP] transposon), eIF4E is required much earlier i.e. already at the 2-cell/4-cell stage. This is supported by the data of the inhibitor 4EG-1, which does not differentate between maternal and paternal source of eIF4E. However, based on allelic discrimination analysis (Fig.S2) the eIF4E transcript is maternal, so the protein synthesized until then must be maternal. Hence the Authors

© 2021. Published by The Company of Biologists under the terms of the Creative Commons Attribution License (https://creativecommons.org/licenses/by/4.0/).

can say that 4EG-1 inhibited the maternal eIF4E; I'm fine with that. The embryos treated with 4EG-1 stop at the 2-cell stage when the treatment starts at the zygote stage, and stop at the 4-cell stage when the treatment starts at the 2-cell stage (Fig.2F,G); whereas fertilization per se is not affected by presence of the inhibitor. This could, in my opinion, be consistent with an effect of eIF4E on the synthesis of cyclins (this possibility is documented in the literature and the Authors have cited one of the relevant studies, Culjkovic et al., 2005). I think this possibility should be acknowledged: our first thought goes to a role of eIF4E in the oocyte-to-embryo transition (lines 390-395), but there is also a remote/trivial possibility whereby the embryos arrest at the 2-cell stage 'simply' because of a cyclin-related problem (this would be consistent with the shift, i.e. embryos arrest at the 4-cell stage if 4EG-1 is administered at the 2-cell stage). This alternative - albeit less interesting -possibility should be mentioned, for the sake of providing the complete picture.

When the 4EG-1 inhibitor was not used, most of the Eif4e-/- embryos from the +/- intercross formed morphological blastocysts. This is probably due to a proportion of maternal protein which was not degraded during cleavage. It can be seen in Figure 3C that the -/- embryos still have some 15-20% residual Eif4e protein. This brings me to my next remark.

The Authors imply that Act-RFP is degraded via the ubiquitin/proteasome system (line 72, and rebuttal) like many other proteins including eIF4E. This is exemplified in Fig.2E (44 h). However, Fig. 1A,B,C shows that the endogenous eIF4E is present all the time. I assume the embryos used for Fig. 1A,B,C were all wildtype. It would be interesting to see what happens with the endogenous eIF4E in the RFP-negative embryos of Fig.2E (44 h): if they degrade eIF4E when they degrade RFP. I understand the breeding issues and how these are exacerbated in Covid-times, therefore I do not ask to perform any experiments. However, there seems to be a discrepancy between what we see with RFP and what we see with the anti-eIF4E antibody, and this should be written in the manuscript. In general, one has to be very careful not to engender a misperception (on the part of the reader) that Act-RFP allows to faithfully monitor the behavior of the endogenous eIF4E locus. I'm sure this distinction is very clear in the Authors' mind, but I am less sure about the readers (including myself). I frankly do not quite see that the statements at lines 194-195 ("The results showed that eIF4E is a maternal product that is lost from the embryo progressively after fertilization. It then becomes progressively re-expressed from the late 2-cell stage…") are supported by the current evidence, unless you would take the embryos that became RFP-negative at 44 h, and show by WB that RFP and eIF4E were both depleted. Somehow, the mutant embryos bother to degrade the RFP, although the eIF4E may still be there. For this reason, given the partial correspondence between RFP and endogenous eIF4E protein, I think that more caution is advised in the wording of the section on protein synthesis (line 263). I trust/accept that the embryos derived from eIF4E +/- intercrosses have lower levels of protein synthesis compared to embryos derived from wildtype crosses (please show image intensities in a histogram), but given Fig.2E and the different RFP behavior of embryos collected fresh from oviduct vs. embryos cultured in vitro, I think the Authors should acknowledge that there are reasons for caution.

The above is meant to help the message come across more clearly to the readers.

Line 211: "all transcripts of materlal origin", the verb (are/were) is missing

Line 392: "It's presence at the critical", should be "Its presence"

All in all, I thank the Authors for the important insights offered by their study into the regulation of the maternal-to-embryonic transition, for the very diligent revision, and I welcome this contribution to the field, pending some final textual amendmends.

Reviewer 2

Advance summary and potential significance to field

This is a revision

Comments for the author

© 2021. Published by The Company of Biologists under the terms of the Creative Commons Attribution License (https://creativecommons.org/licenses/by/4.0/).

The authors addressed some of the concerns raised by the reviewers, but some important criticisms were not addressed, or addressed unsatisfactorily, because of the difficulties caused by the covid-19 pandemic. Some experiments must be redone (Fig.1A), and statements need to be changed because of lack of data, as follows:

1. In response to the reviewer's #2 request (point #5), the authors added the ACTIN loading control. However, Figure 1A is still unacceptable as the western blot for eIF4E, and p-eIF4E originated from separate gel runs. Based on the shape of the ACTIN control bands it is from the same gel as for total eIF4E. Thus, the p-eIF4E still lacks a loading control. It is needless to say that the blotting for eIF4E and p-eIF4E must be from the same gel.
Also, it is very unusual to show a western blot without molecular weight markers. Since the unphosphorylated eIF4E migrates slower (Molecular mass of 33 kDa) than the p-eIF4E (Molecular mass of 28 kDa), which is contrary to the existing literature, it is incumbent upon the authors to show the molecular weight markers.
2. They must quantify the data in Fig. 4, particularly Fig. 4E since in the text they claim the OPP incorporation is higher in eIF4E+/+ x eIF4E+/+.
3. In light of the pandemic-related inability to confirm the results of Fig. 6D with a Western blot they ought to remove panels that show "Blocking pf the phosphorylation of 4E-BP1 and S6K1 by 4EGI-1".

Second revision

Author response to reviewers' comments

Reviewer 1 Advance Summary and Potential Significance to Field:

Reviewer 1 Comments for the Author:

This could, in my opinion, be consistent with an effect of eIF4E on the synthesis of cyclins (this possibility is documented in the literature and the Authors have cited one of the relevant studies, Culjkovic et al., 2005). …. This alternative - albeit less interesting -possibility should be mentioned, for the sake of providing the complete picture.

Thank you for this useful suggestion we have now added some commentary on this point to the discussion in the revised MS "*The analysis of translation in the early embryo was consistent with the expected roles of eIF4E in its initiation. The study does not show what products of translation are required at the critical stages for further development. The developmental blocks resulting from the early pharmacological inhibition of eIF4E may reflect the requirement of translation of critical components of the mitotic machinery, such as the cyclins (Groisman et al., 2002; Culjkovic et al., 2005), or components of the transcriptional apparatus required for EGA (e.g. new protein synthesis of activating transcription factor (ATF1) was required for zygote maturation (Jin and O'Neill, 2014).*" - P14, Line382- 389

The Authors imply that Act-RFP is degraded via the ubiquitin/proteasome system (line 72, and rebuttal) like many other proteins including eIF4E. This is exemplified in Fig.2E (44 h). However, Fig. 1A,B,C shows that the endogenous eIF4E is present all the time. I assume the embryos used for Fig. 1A,B,C were all wildtype. It would be interesting to see what happens with the endogenous eIF4E in the RFP-negative embryos of Fig.2E (44 h): if they degrade eIF4E when they degrade RFP. I understand the breeding issues and how these are exacerbated in Covid-times, therefore I do not ask to perform any experiments.
However, there seems to be a discrepancy between what we see with RFP and what we see with the anti-eIF4E antibody, and this should be written in the manuscript.

We agree that this is an important anomaly. This may arise due to difference in the manner or kinetics of how cells deal with RFP compared to the native protein - eIF4E. We agree that it is

© 2021. Published by The Company of Biologists under the terms of the Creative Commons Attribution License (https://creativecommons.org/licenses/by/4.0/).

important to make the point that RFP may not show the exact same temporal profile of expression as the native protein and have tried to reinforce this point further in the revised discussion- "*The rapid loss RFP after fertilization likely reflects a combination of the transcriptionally inert state of the early zygote and the onset of proteolytic degradation of many maternally-inherited proteins after fertilization. However, it is important to consider that the kinetics of the degradation of RFP may not reflect that of native eIF4E since there are many aspects of individual protein structure that influence this kinetic (Grumati and Dikic, 2018; Tsukamoto and Tatsumi, 2018; Toralova et al., 2020). It is also possible that the kinetics of protein degradation vary depending on whether embryos are collected directly from the reproductive tract or are cultured in vitro. Given the nature of the experimental design it was not possible in this study to control for these variables.*" (P13 Line349-3567) and **results Page 11 Line 289-292 We have inserted "*It is important to recognize that this method requires that embryos are cultured in vitro for varying periods and the model does not allow us to assess any potential interactions between the genetic model and any adverse effects of the culture procedures.*"**

Line 211: "all transcripts of maternal origin", the verb (are/were) is missing
A; Edited

Line 392: "It's presence at the critical", should be "Its presence"
A; Edited

Reviewer 2 Advance Summary and Potential Significance to Field:
This is a revision

Reviewer 2 Comments for the Author:
1. In response to the reviewer's #2 request (point #5), the authors added the ACTIN loading control. However, Figure 1A is still unacceptable as the western blot for eIF4E, and p-eIF4E originated from separate gel runs. Based on the shape of the ACTIN control bands it is from the same gel as for total eIF4E. Thus, the p-eIF4E still lacks a loading control. It is needless to say that the blotting for eIF4E and p-eIF4E must be from the same gel.

Also, it is very unusual to show a western blot without molecular weight markers. Since the unphosphorylated eIF4E migrates slower (Molecular mass of 33 kDa) than the p-eIF4E (Molecular mass of 28 kDa), which is contrary to the existing literature, it is incumbent upon the authors to show the molecular weight markers.

A: We have now added an example of the an original gel which show the showing the size of of eIF4E, p-eIF4E and ACTIN after stripping and re-probing same gel (Fig 1A), on Page 4, line 118-121. Insert the description "*We first established a method for the simultaneous quantitative detection of eIF4E and phosphorylated eIF4E (p-eIF4E) relative to the levels of ACTIN in embryos by Western blot analysis. Clear signals for all three antigens could be detected in groups of 50 zygotes with little variability in signal strength between samples (Fig 1A)*" and then analyzed the relative quantity of both eIF4E and p-eIF4E relative to ACTIN from gel prepared Fig 1 B. Also, we edited relevant description on methods, result and figure legend to provide more detail of the methods labelling and quantification. (Page 20, Line 565-574, Page21-22, Line 606-615).

2.They must quantify the data in Fig. 4, particularly Fig. 4E since in the text they claim the OPP incorporation is higher in eIF4E+/+ x eIF4E+/+.

A: We measured the OPP intensity of whole embryos and add the results in Figure 4 F and G, edited the manuscript (Page 10-11, Line 280-288) "*Embryos that showed developmental delay by arrest prior to the expected 8-cell stage had the highest levels of RFP and a corresponding lower capacity for translation, as assessed by OPP staining intensity (Fig 4E, F). The overall level of protein expression was lower in embryos from Eif4e+/- cross-mating than the embryos that derived from Eif4e+/+ mating, but this was solely due to embryos that had retarded development and were likely to be either homozygous or heterozygous for the transgene,*"

© 2021. Published by The Company of Biologists under the terms of the Creative Commons Attribution License (https://creativecommons.org/licenses/by/4.0/).

judged by the high levels of RFP expression in these embryos. Embryos from Eif4e+/- crosses that achieved their expected developmental landmark of the 8-cell stage had significantly lower levels of RFP than retarded embryos and had similar levels of translation (OPP expression) as equivalent embryos from Eif4e+/+ crosses (Fig 4G)".
We provide the detail of how this quantitative analysis was performed in the methods (P17, line 472-475) and figure legend (Page 23-24, Line 6663-671) section and also relevant description on the statistical analyses.

3. In light of the pandemic-related inability to confirm the results of Fig. 6D with a Western blot they ought to remove panels that show "Blocking pf the phosphorylation of 4E-BP1 and S6K1 by 4EGI-1".
A: We have edited the figure 6 as suggested. And removed the statement from manuscript.

Third decision letter

MS ID#: DEVELOP/2020/190793

MS TITLE: The regulation of the mammalian maternal-to-embryonic transition by Eukaryotic translation initiation factor 4E

AUTHORS: Yan Li, Jianan Tang, Xu JI, Min-Min Hua, Miao Liu, Lu Chang, Yihua Gu, Changgen Shi, Wuhua Ni, Jing Liu, Hui-juan Shi, Xuefeng Huang, Christopher O'Neill, and Xingliang Jin
ARTICLE TYPE: Research Article

Your response to the review and the revision of the manuscript are satisfactory. I am happy to tell you that the paper has been accepted for publication in Development, pending our standard ethics checks.

© 2021. Published by The Company of Biologists under the terms of the Creative Commons Attribution License (https://creativecommons.org/licenses/by/4.0/).
